# Evaluation of Inhalation Exposures and Potential Health Impacts of Ingredient Mixtures Using *in vitro* to *in vivo* Extrapolation

**DOI:** 10.3389/ftox.2021.787756

**Published:** 2022-02-02

**Authors:** Jingjie Zhang, Xiaoqing Chang, Tessa L. Holland, David E. Hines, Agnes L. Karmaus, Shannon Bell, K. Monica Lee

**Affiliations:** ^1^ Altria Client Services, LLC, Richmond, VA, United States; ^2^ Integrated Laboratory Systems, LLC, Morrisville, NC, United States; ^3^ Lancaster Laboratories, c/o Altria Client Services, LLC, Regulatory Affairs, VA, Richmond, United States

**Keywords:** new approach methodologies (NAMs), electronic cigarette, *in vitro* to *in vivo* extrapolation (IVIVE), physiologically based pharmacokinetic (PBPK) model, *in vitro* toxicity mechanism, mixture assessment

## Abstract

*In vitro* methods offer opportunities to provide mechanistic insight into bioactivity as well as human-relevant toxicological assessments compared to animal testing. One of the challenges for this task is putting *in vitro* bioactivity data in an *in vivo* exposure context, for which *in vitro* to *in vivo* extrapolation (IVIVE) translates *in vitro* bioactivity to clinically relevant exposure metrics using reverse dosimetry. This study applies an IVIVE approach to the toxicity assessment of ingredients and their mixtures in e-cigarette (EC) aerosols as a case study. Reported *in vitro* cytotoxicity data of EC aerosols, as well as *in vitro* high-throughput screening (HTS) data for individual ingredients in EC liquids (e-liquids) are used. Open-source physiologically based pharmacokinetic (PBPK) models are used to calculate the plasma concentrations of individual ingredients, followed by reverse dosimetry to estimate the human equivalent administered doses (EADs) needed to obtain these plasma concentrations for the total e-liquids. Three approaches (single actor approach, additive effect approach, and outcome-oriented ingredient integration approach) are used to predict EADs of e-liquids considering differential contributions to the bioactivity from the ingredients (humectant carriers [propylene glycol and glycerol], flavors, benzoic acid, and nicotine). The results identified critical factors for the EAD estimation, including the ingredients of the mixture considered to be bioactive, *in vitro* assay selection, and the data integration approach for mixtures. Further, we introduced the outcome-oriented ingredient integration approach to consider e-liquid ingredients that may lead to a common toxicity outcome (e.g., cytotoxicity), facilitating a quantitative evaluation of *in vitro* toxicity data in support of human risk assessment.

## Introduction

Electronic cigarettes (EC) are gaining popularity among adult smokers who are looking for reduced-risk alternatives. Since EC are noncombustible and have substantially lower levels of harmful and potentially harmful constituents (HPHCs) they hold the promise as potentially reduced-harm alternative to combustible tobacco products. However, its long-term health effects are currently unknown. The e-liquid and the produced aerosol are both complex mixtures, which are typically composed of flavor ingredients, nicotine, organic acids, and carrier chemicals (propylene glycol [PG] and vegetable glycerol [VG]). PG and VG are commonly used carriers in the EC products and make up a large portion of the e-liquid (e.g., approximately 80% of the total mass and above, National Academies of Sciences, Engineering, and Medicine. 2018). Nicotine is considered the active ingredient in the e-liquid while flavors are added to accommodate user’s sensory preference. Hundreds of unique flavor ingredients are identified based on reviews of e-liquids on the market (Hua et al., 2019; Krüsemann et al., 2021; Salam et al., 2020), with acids (such as lactic, benzoic, levulinic, salicyclic, malic, and tartaric acids) in some EC products converting nicotine to nicotine salts (Harvanko et al., 2020). The combinations of these ingredients lead to a myriad of e-liquids in the market. Traditionally, the health impact of EC products is evaluated as ingredients or as a whole product mixture in preclinical *in vitro* and if necessary *in vivo* testing, with carriers at various PG:VG ratios as the control (Merecz-Sadowska et al., 2020). Currently, data from *in vivo* animal testing is often regarded the gold standard for risk assessment, although to test all the EC products is unrealistic and against the current move to 3R’s principle (refinement, reduction, and replacement) (Russell and Burch, 1959; FDA, 2019), considering the number of studies and animals needed. In addition, *in vivo* data also have inherent uncertainty of the interspecies extrapolation from animal to human.

New approach methodologies (NAMs) such as human cell-based *in vitro* testing can potentially provide a rapid approach to assess hazards and toxicity potential to support risk assessments, while reducing or eliminating the need for animal testing. *In vitro* testing can provide various stages of mechanistic information about the bioactivity of test compounds without traditional descriptive animal testing. However, most current applications of *in vitro* testing within safety assessment are for screening, prioritization and hazard identification rather than quantitative risk assessment. One of the challenges in using NAMs for chemical risk assessment is to relate *in vitro* bioactivity dose-responses to relevant *in vivo* exposures (Parish et al., 2020). To put the *in vitro* data into *in vivo* context requires the use of *in vitro* to *in vivo* extrapolation (IVIVE) approaches. The IVIVE approach uses physiologically based pharmacokinetic (PBPK) models to generate in silico predictions of exposure metrics in human based on *in vitro* data. Specifically, PBPK models predict the amount of chemical reaching systemic circulation or tissues of interest corresponding to biological responses measured with *in vitro* assays. The IVIVE approach then uses PBPK models to translate *in vitro* bioactivity data to corresponding equivalent administered doses (EADs) *in vivo*, thus estimating an external dose in humans that may induce similar bioactivity reflected by the *in vitro* assay (Bell et al., 2018). Fit-for-purpose IVIVE analyses require consideration of several key factors, including pharmacokinetic modeling, biochemical and biophysical properties of the test article, *in vivo* (human) exposure parameterization, and the selection of *in vitro* assays (Bell et al., 2018; Chang et al., 2021). Several case studies have explored these fit-for-purpose modeling approaches for individual chemicals (Clewell et al., 2008; Yoon et al., 2014; Baltazar et al., 2020; Algharably et al., 2021). However, there are few studies that investigate the application of the IVIVE approach for mixtures.

There are several challenges when conducting computational modeling of mixtures (Raies and Bajic, 2016) including IVIVE analysis. One challenge is that the *in vitro* testing for the mixture is sometimes designed to assess the entirety of the mixture while PBPK model and parameterization is, by default, constructed for specific individual compounds. In e-liquids and EC aerosols, the differential pharmacokinetics of the mixture ingredients may also shift the availability of those ingredients in plasma compared to the exposure condition employed in the *in vitro* testing. In addition, each ingredient in the mixture may have different modes of action (MOA) and may induce perturbations in multiple, disparate biological pathways that may or may not lead to the same *in vitro* or *in vivo* outcomes. Moreover, there may be agonistic and antagonistic interactions among ingredients, as well as phase partitioning in multiphase flows (e.g., tobacco smoke and e-vapor aerosols).

Given that most exposures of interest are as mixtures rather than single chemicals, there have been many efforts trying to address the above challenges through modeling (Desalegn et al., 2019). Expressing the mixture components in a standardized way and treating the exposure additively is one common approach (Delistraty, 1997; Haddad et al., 2001). Other studies that have looked at how to integrate additional information to address the toxicity as well as the absorption, dispersion, metabolism and excretion (ADME) of the mixture (Ruiz et al., 2020). No standardized approaches are currently available for computational modeling of mixtures. Therefore, it is necessary to define and evaluate fit-for-purpose criteria to guide the calculation for the mixture IVIVE modeling.

In a previous case study (Chang et al., 2021), IVIVE analyses were conducted for the exposure and health impacts of nicotine and flavor mixtures in EC products using publicly available *in vitro* cytotoxicity data of the mixtures (Omaiye et al., 2019) and *in vitro* bioactivity for individual ingredients available from the ToxCast/Tox21 inventory. Chang et al. (2021) provide a methodology to predict maximal plasma concentrations (Cmax) for ingredients and to integrate ingredient-level Cmax predictions to generate product-level estimates based on both individual ingredient and mixture bioactivity. As a proof of concept, this simplified methodology assumes minimum interaction between ingredients, equal time to reach the Cmax in the plasma after exposure (Tmax) across ingredients, and additive effects of accounted ingredients in the mixtures. It also assumes that the biological responses of mixtures are attributed only to nicotine and flavors that are identified and quantitated in the e-liquids and aerosols as reported in Omaiye et al. (2019), which did not include other major ingredients (PG, VG and benzoic acid [BA]). However, PG, VG and BA are major ingredients in the tested e-liquids and aerosols and their *in vitro* bioactivity is demonstrated in other studies (Sassano et al., 2018; Ghosh et al., 2021). Understanding how the results are affected by the composition of the test mixture is necessary for applying IVIVE analyses to inform decision making towards risk assessment.

In this work, we expand Chang et al. (2021) case study on the application of IVIVE for mixtures, with the following specific goals: 1) to understand the contribution of the carriers and acid to the EAD predictions when their *in vitro* activity is considered and 2) to develop and evaluate methods by which the ingredient-level data are integrated to better inform mixture bioactivity. We achieved the two goals in two stages described as below. The first stage predicted EADs using *in vitro* bioactivity data of e-liquid mixtures and evaluated the impact of the contribution of carriers and the acid to the EAD predictions by comparing the results to the previous study (Chang et al., 2021). The second stage focused on integrating ingredient-level *in vitro* bioactivity information to provide insight on the bioactivity of the mixtures and discussed the selection of ingredient-level *in vitro* assays based on cytotoxicity. The ingredient and mixture data were obtained from publicly available sources, and an open-source, generic PBPK model was used to perform the EAD estimations. Methods are developed to facilitate the mixture integration and described.

## Materials and Methods

### E-Liquid Composition

Publicly available data of the e-liquid composition were obtained (Omaiye et al., 2019). Briefly, we extracted the numerical flavor and nicotine concentrations for the eight e-liquids from figures in Omaiye et al. (2019) using WebPlotDigitizer (Rohatgi, 2019) as described in Chang et al., 2021. As carrier ingredients (PG and VG) and the BA were not analytically quantified in Omaiye et al. (2019), we estimated their concentrations using the reported nicotine concentration and PG:VG ratio of 30:70 by mass (https://www.fda.gov/tobacco-products/market-and-distribute-tobacco-product/deemed-new-tobacco-product-applications-lists#list%20of%20deemed; checked on December 01 2021), assuming BA in equal molar concentration to nicotine. Estimated concentrations of all ingredients of the eight e-liquids in this study are presented in Supplementary Table S1.

### *In vitro* Bioactivity Data

*In vitro* cytotoxicity data for the EC aerosols were obtained from the dimethylthiazol diphenyltetrazolium bromide (MTT) and neutral red uptake (NRU) assay data in Omaiye et al. (Omaiye et al., 2019). Both MTT and NRU assays provided the half-maximal inhibitory concentration (IC50) values as indicators of cellular cytotoxicity (Omaiye et al., 2019); IC50 values are included in the Supplementary Table S1.

*In vitro* bioactivity data for single ingredients were obtained from the curated HTS assays available from the Integrated Chemical Environment (ICE; Bell et al., 2017; Bell et al., 2020), which provides a curated version of the U.S. EPA’s invitroDB V3.2 (accessed December 2020) taking into account chemical quality control information. Furthermore, an additional manual review of the concentration-response curves was performed to remove any ambiguous bioactivity calls to improve robustness of the final dataset. Among the 46 ingredients which were identified and quantitated across the eight e-liquids (Omaiye et al., 2019), information was not available for 13 ingredients (not tested) with an additional 12 having no active assay data passing curation. This resulted in 21 out of the 46 ingredients across all e-liquids with data for subsequent analyses. The ingredient AC50 of the 21 ingredients are available in Supplementary Table S2 with values used for the outcome-oriented integration approach in Supplementary Table S3.

### PBPK Models

We used an open-source eight-compartment PBPK (physiologically based pharmacokinetic) model for inhalation exposure (Gas_PBTK) from the httk (High-Throughput Toxicokinetics) package to calculate the Cmax resulting from each ingredient (Pearce et al., 2017). A 1 mg/kg single daily dose was used for determining plasma Cmax for conducing the IVIVE analysis. The gas PBPK model uses an inhaled air concentration (uM); therefore, a unit conversion from mg/kg to uM was conducted assuming complete aerosolization of the e-liquid and uniform dispersion in air for an inhaled chemical. The airborne concentration was obtained by dividing the total chemical mass (mg) by the total inhaled air volume over an assumed exposure period (15 min). The total inhaled air volume (L) is calculated by multiplying average human tidal volume (0.6 L/breath), respiratory rate (12 breath/minute) and the 15-min exposure period (Needham et al., 1954; Russo et al., 2017). The air concentration (mg/L) was converted to uM using the molecular weight prior to use as dosing input for Gas_PBTK model. The plasma Cmax at a dose of 1 mg/kg of each ingredient at 2-h dosing intervals was also predicted and the results are available in Supplementary Table S5. The impact of the 2-h and 24-h dosing regimens were discussed in the previous study (Chang et al., 2021).

Parameters used in PBPK modeling for each ingredient were obtained from ICE (accessed March 2021). Modeling was limited to the ingredients only and did not consider metabolites or potential byproducts from interactions. When available, measured values for hepatic intrinsic clearance (Clint) and fraction unbound (fu) were used for ingredients. Otherwise, in silico predictions were used. Physiochemical data was obtained from OPERA (v2.6) QSAR models and used to calculate additional parameters using internal functions of the httk package (Pearce et al., 2017). Parameters used in modeling along with the sources are provided in Supplementary Table S4. All other parameters used the httk’s internal values or calculated valued based on these provided parameters.

### Approaches for the Estimation of Human EADs

We applied IVIVE to estimate EADs using three approaches: the single actor approach (for EC aerosols and ingredients), the additive effect approach (for EC aerosols), and the outcome-oriented ingredient integration approach (for ingredients). The first two approaches are described in Chang et al. (2021). In this study, we developed the outcome-oriented ingredient integration approach to allow “combining” or integrating *in vitro* bioactivity data from multiple ingredients in a mixture based on a common biological target or process, in contrast to the single actor approach. In the outcome-oriented ingredient integration approach, we select a representative assay data for each ingredient and estimate the combined EAD for the targeted biological responses. Data can be integrated from different assays resulting in the same changes at the molecular level or leading to the same biological outcome (e.g., cytotoxicity) creating a better estimate of the additive effect the ingredients may have. The outcome-oriented ingredient integration approach is similar to the additive effect approach used for the e-liquid mixtures but using the individual ingredient *in vitro* (HTS) data as opposed to the mixture *in vitro* (MTT) data. In this study we used *in vitro* cytotoxicity data as the common target for the integration.

#### Single Actor Approach

The single actor approach assumes the observed *in vitro* activity of a mixture is solely attributable to a single ingredient in the mixture. For each single ingredient that is selected as the “single actor,” an EAD for the mixture is estimated based on the *in vitro* activity concentration (AC) of that ingredient. This approach was used for calculating the EAD of the e-liquid from mixture cytotoxicity data as well as from HTS data for individual ingredients.

To calculate the single actor EAD for the EC aerosol, we first calculate the EAD for each ingredient, which is then divided by mass fraction of the ingredient. To calculate the EAD for each ingredient (Equation 1), the IC50 for the mixture was first adjusted by the mass fraction of the ingredient to get the IC50 for the ingredient, which was then divided by the maximum plasma concentration (Cmaxi) at a dose of 1 mg/ kg. When considering only single ingredient activity data, the lowest AC50 values of *in vitro* HTS data was used. The Equation 1EAD_i_ Eq. 1 is calculated by dividing the lowest AC50 by Cmax at a dose of 1 mg/kg and then scaling with the mass fraction of the ingredient in the mixture to get EAD for the mixture.

$$EAD_{mix-i}=\frac{EAD_{i}}{frac_{i}}=\frac{\frac{AC_{i}}{Cmax_{i}}}{frac_{i}}=\frac{AC_{total}*\frac{frac_{i}}{Cmax_{i}}}{frac_{i}}=\frac{AC_{total}}{Cmax_{i}}$$ (1)

(Where AC is the activity concentration of a mixture)

$$EAD_{mix-i}=\frac{EAD_{i}}{frac_{i}}=\frac{AC_{i}/Cmax_{i}}{frac_{i}}$$ (2)

(Where AC is the activity concentration of a single chemical).

In Eqs 1, 2, EAD_mix-i_ is the equivalent administered dose for the mixture estimated based on *in vitro* activity concentration of chemical *i,* mg/kg; EAD_i_, is the EAD for chemical *i* corresponding to *in vitro* activity concentration of chemical *i, mglkg;* frac_i_ is the mass fraction of chemical *i* in the mixture; AC_i_ is activity concentration for an ingredient from *in vitro* assay. It is IC50 from the *in vitro* cytotoxicity assay of EC aerosol mixture after being adjusted by the mass fraction of chemical *i* (Eq. 1) or AC50 from *in vitro* assay of an ingredient (Eq. 2); AC_total_ is the activity concentration of *in vitro* cytotoxicity assay of EC aerosol; Cmax_i_ is the maximum plasma concentration at 1 mg/ kg/ dose of Eq. 1 chemical *i;* For additional context, EADs were converted to e-liquid pod equivalents by dividing the total mass of a single pod after being scaled up to whole body exposure (Supplementary Figures S1–S3).

#### Additive Effect Approach

The additive effect approach assumes all chemicals in the mixture contribute to the *in vitro* bioactivity of the mixture proportionally to their mass fraction in the mixture. This creates a single point estimate of the EAD-mix representing the integration of the activities. The *in vitro* cytotoxicity data of the mixtures (MTT, Omaiye et al., 2019) were also used in this approach. To compare the impact of including the total ingredients in EC product as contributing to bioactivity, as opposed to previous Chang et al., we considered the IC50 value representing either the bioactivity from the quantified ingredients (nicotine and flavor; Chang et al., 2021) or the total product (nicotine, flavors, BA and carriers).

When only a subset of ingredients is used as in Chang et al. (nicotine and flavors), the equation to calculate the EAD and number of pods is as follows:

$$EAD_{mix}=\frac{AC_{total}*\sum_{i=1}^{m}frac_{i}}{\sum_{i=1}^{m}\left(Cmax_{i}*frac_{i}\right)}$$ (3)

In Equation 3, EAD_mix_ is the equivalent administered dose for the mixture, AC_total_ is the IC50 of *in vitro* cytotoxicity assay of the EC aerosol, Cmax_i_ is the maximum plasma concentration at 1 mg/kg/dose of ingredient *i*, frac_i_ is the fraction of individual flavor or nicotine ingredient in the total product, and m is the number of ingredients in the subset.

When all ingredients of the e-liquid (total product) are assumed to contribute to the bioactivity, the equation to calculate the EAD is as follows:

$$EAD_{mix}=\frac{AC_{total}}{\sum_{i=1}^{n}\left(Cmax_{i}*frac_{i}\right)}$$ (4)

Equation 4 uses the same variable names as Eq. 3 except that n is the total number of ingredients in the e-liquid (total product). As with the single actor analysis, we provided additional context for the EAD predictions by converting them to e-liquid pod equivalents dividing the mass of a single pod (0.7 ml) scaled for body weight (70 kg) (Supplementary Figures S1, 2).

#### Outcome-Oriented Ingredient Integration Approach

The outcome-oriented ingredient integration approach quantitatively integrates individual chemical bioactivity measures from *in vitro* HTS assays to provide estimates of the mixture bioactivity with common biological responses. This approach assumes that chemicals affecting the same targeted outcome contribute in an additive manner and the Cmax from each chemical occurs at the same time (i.e. Tmax is the same). While it is likely different chemicals in a mixture have different Tmax values, this simplification can provide a conservative estimate as it maximizes the total concentration (Cmax). In this study, we selected cytotoxicity or cell viability as the common biological response and used relevant assays from the HTS database, which is parallel to the MTT and NRU assays used in EC mixtures. ICE provided mapping of the HTS assays to mechanistic targets (Bell et al., 2020) and was used to identify assays annotated to cytotoxicity (cell viability) in the integrated analysis (Supplementary Table S2). The majority of the ingredients tested in the cell viability assays were not found to be bioactive (inactive designation). Ingredients with at least one active HTS cell viability assay (7 ingredients, out of total 21) were designated as active for the purposes of this analysis, as most of the cell viability assays for these ingredients were inactive (Table 1).

**TABLE 1:** Ingredients treated as active in the integration analysis based on at least one active HTS cytotoxicity assay.

CASRN	Ingredient name	Number of cytotoxicity assays	Number active assays	AC50 for integration approach; geometric mean^a^ (uM)
104-50-7	4-Octanolide	66	1	383.3755
104-67-6	5-Heptyldihydro-2(3H)-furanone	81	2	380.1397
562-74-3	4-Methyl-1-(propan-2-yl) cyclohex-3-en-1-ol	66	1	390.8753
706-14-9	gamma-Decanolactone	66	1	380.6671
57-55-6	1,2-Propylene glycol	81	1	386.8474
87-25-2	Ethyl anthranilate	68	11	274.1430
99-49-0	dl-Carvone	66	1	389.1948

a Geometric mean of AC50 values was calculated using measured AC50 values and a value of 400 uM for inactive assay results.

To account for this distribution of active and inactive assays, the geometric mean of AC50 values was calculated to provide a weighted bioactivity estimate considering both active and inactive assays for each ingredient. Inactive assay results were assigned an AC50 value of 400 uM, twice the maximum concentration tested (200 uM) for most ingredients. Ingredients that were tested in HTS cell viability assays but had all inactive results (26 ingredients) were considered inactive in this analysis (designated with 400 uM). It is notable that three major EC ingredients (nicotine, BA, and VG, making up about 30% of total mass) were inactive for Tox21/ToxCast HCS cytotoxicity assays (Table 1). When no cytotoxicity data were available for an ingredient (13 ingredients), the geometric mean AC50 value (387.012 uM) across all cell viability assays, calculated considering both the active and inactive (400 uM) response values as a conservative estimate. Full details about the number of assays tested for each ingredient and the AC50’s used in the integration analysis are shown in Supplementary Table S3. An RNotebook with code describing the analysis is available in Supplementary File S1.

The integration approach first predicted plasma concentrations for each ingredient and adjusted them based on the ingredient’s mass percent in the mixture. The “Gas_PBTK” httk model was used for Cmax prediction. Then, using AC50 data as a measure of bioactivity for each active or not tested ingredient (Supplementary Table S3), individual ingredient plasma concentrations were scaled to “equivalent plasma concentration” of the most sensitive mixture ingredient. These equivalent plasma concentrations were calculated using the ratio of the ingredient AC50 to the lowest AC50 and describe the Cmax of the most sensitive ingredient that is expected to contribute the same bioactivity as the predicted Cmax for the focal ingredient. Finally, these relative plasma concentrations for each mixture ingredient were summed to predict activity from the mixture. A detailed example calculation is provided in Data Sheet S2 to demonstrate each step of the analysis using a hypothetical mixture.

We compared the EAD for *in vivo* toxicity (based on cytotoxicity *in vitro*) from two different IVIVE scenarios: “total product,” and “flavor only.” The total product scenario contained all active or not tested ingredients in the mixture, while the flavor only scenario considered only flavor ingredients (Supplementary Table S3). Ingredient Cmax and AC50 values of included ingredients were used to estimate EAD for each EC flavor under both scenarios.

## Results

### EAD Predictions Using the Cytotoxicity Data of EC Aerosols

**FIGURE 1:**
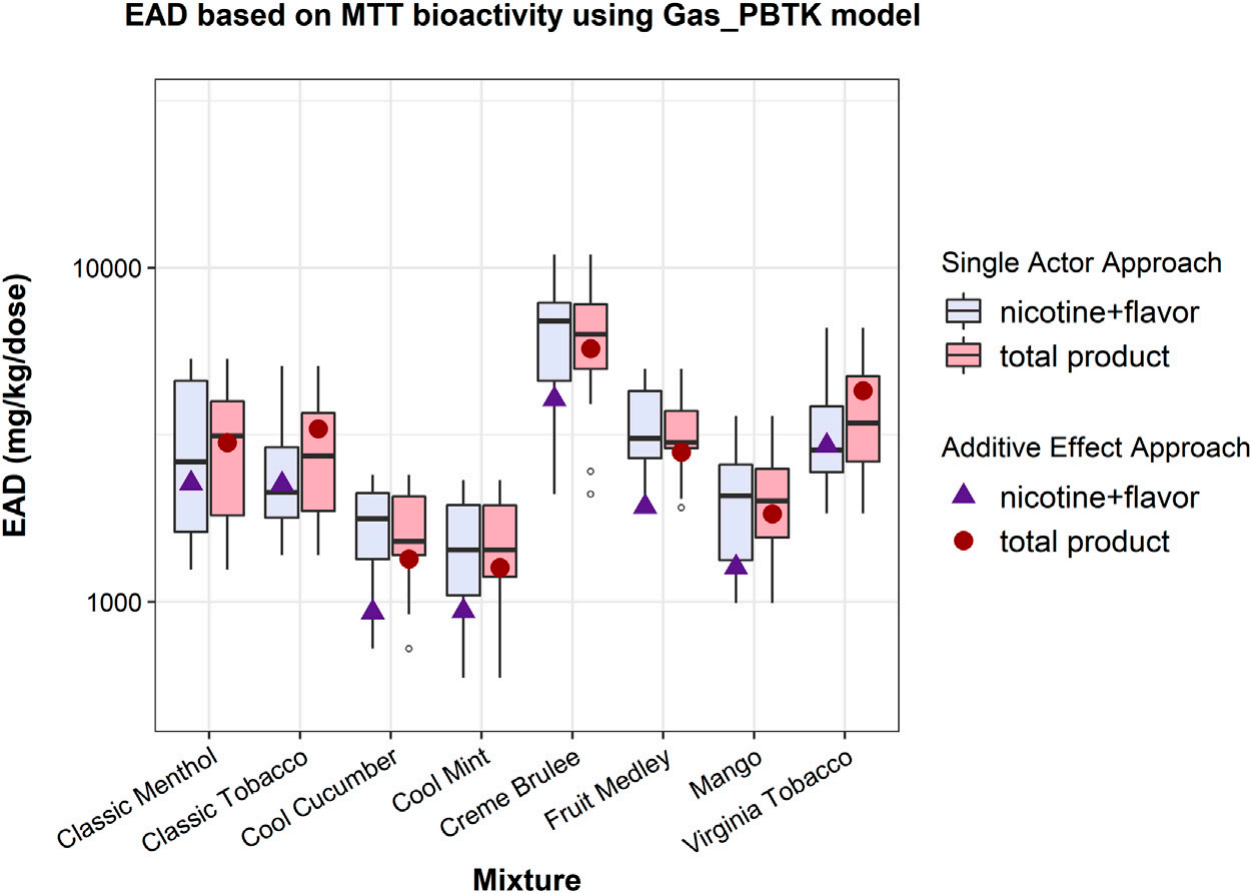
EAD estimates for the bioactivity of e-liquids based on the MTT assay using different assumptions about contributors to bioactivity under both the single actor and additive approaches. “Total product” (shown in red) assumes all e-liquid ingredients (including PG, VG, and BA) contribute to bioactivity, while “nicotine + flavor” (shown in blue) assumes only nicotine and flavors contribute to bioactivity. Box plots show distributions of results across all ingredients using the single actor approach while points (solid triangles and circles) show the point estimate for EAD using the additive effect approach.

Both the single actor and additive effect approaches (Chang et al., 2021) were applied to estimate EADs from the MTT data of EC aerosols (Omaiye et al., 2019). With the single actor approach, comparison of EADs from “total product” and “nicotine + flavor” showed that the inclusion of PG, VG, and BA (total product) did not change the upper or lower limits of the EAD range while the median shifted due to different compositions of each EC aerosol (Figure 1). The distribution of the EAD estimates varied depending on what ingredient was used as the primary contributor to the bioactivity. With the additive effect approach, the inclusion of PG, VG and BA resulted in an increased to the calculated EAD (Figure 1) compared to considering just “nicotine + flavor”. This is due to a combination of the ADME properties and the percent composition of the ingredients and thus may change if different carrier ingredients were considered. EAD estimations using the NRU assay data (Supplementary Table S1), along with pod exposure estimations can be seen in Supplementary Figures 1, 2.

### EAD From Single Actor Approach Using Ingredient HTS Data

The single actor approach was used to predict EADs for mixtures using the assay with the lowest AC50 (representing the most sensitive assay) *in vitro* HTS data from the ToxCast and Tox21 programs for each ingredient (Supplementary Table S2). This approach considered 21 ingredients out of total 46, which have at least one valid active *in vitro* assay in the HTS database. The annotated mechanisms of these *in vitro* assays include cytotoxicity and various early cellular responses, such as receptor modulation and oxidative stress.

**FIGURE 2:**
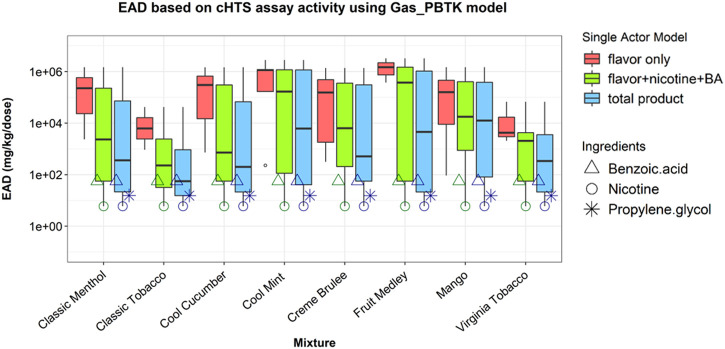
EAD estimated using the HTS data for individual ingredients in the single actor approach. EAD estimates were generated based on each ingredient’s most sensitive bioactivity from the HTS assay and adjusted for the ingredient’s concentration in the e-liquid flavor. The data were plotted based on each of three combinations: flavor, flavor + nicotine + BA, and total product. Boxplots show the distribution of EAD calculations for all considered ingredients using the single actor approach. Triangle, circle, and asterisk points indicate the EADs predicted when BA, nicotine, and PG were used as active ingredients, respectively.

The EAD predictions using the Gas_PBTK model are shown in Figure 2. Comparisons among “flavor”, “flavor + nicotine + BA”, and “total product” showed the impact of nicotine’s bioactivity on IVIVE outcomes. Nicotine, while making up ∼5% of the aerosolized mixture, generated the lowest EAD for combinations where it was included. The AC50 (1.36 µM) used for this estimation is based an *in vitro* assay of which the mechanistic target is annotated as neurotransmission (competitive binding to neuronal acetylcholine receptor subunit α -2). This supports the earlier observation that considering the combination of flavor and nicotine may provide a conservative estimate for bioactivity (Chang et al., 2021). Furthermore, the PG (AC50 of 26.66 µM from a cell viability assay) and BA (AC50 of 7.54 µM from an assay of which the mechanistic target cannot be annotated) consistently produced the second and third lowest EAD estimates (Figure 2) wherever they were included. The median of “flavor only,” “flavor + nicotine + BA” and “total product” ranked from high to low for all eight EC mixtures, indicating increased health risks.

**FIGURE 3:**
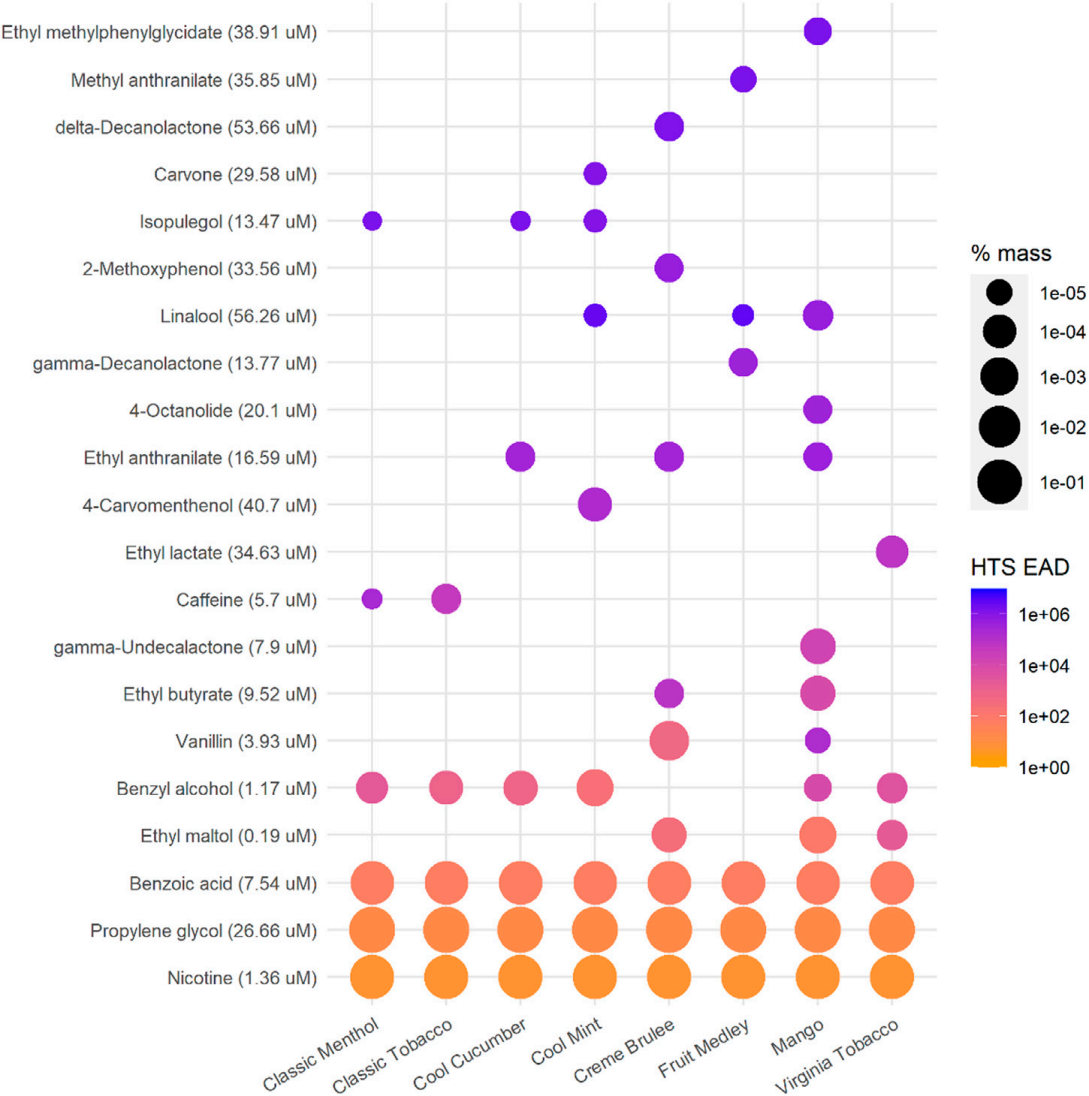
EAD predictions for individual ingredients using the single actor approach and the lowest AC50 values reported in the HTS data sets. Data are ordered based on the minimum EAD for each ingredient across all mixtures. Orange colors indicate lower EAD estimations (more toxic), while blue colors show higher EAD estimations. The size of dots indicates the mass percentage of the e-liquid mixture for each ingredient (log scale). Note that nicotine and BA represent ∼5% mass while PG represents ∼30% mass (Supplementary Table S1). The AC50 for each ingredient used in calculating is shown in parentheses next to the ingredient name.

The EAD predictions for each ingredient in the EC mixtures are shown in Figure 3. Notably ingredients with lower AC50 values did not necessarily produce the lowest EAD predictions, as the mass fraction as well as the metabolic clearance also played a role in EAD estimation using this approach. For example, the lowest available AC50 for isopulegol is 13.47 uM from an *in vitro* assay that measures estrogen receptor modulation, which is about half of the PG AC50 (26.66 µM), but the estimated EAD based on isopulegol was 10,000-fold higher than that estimated based on PG mainly because the percent mass of isopulegol is much lower than PG (10^–4^ *vs*. 10^2^ in terms of order of magnitude) in the formulations containing both ingredients (Figure 3; Supplementary Table S1).

### EAD From Outcome-Oriented Ingredient Integration Approach Using Ingredient HTS Data

**FIGURE 4:**
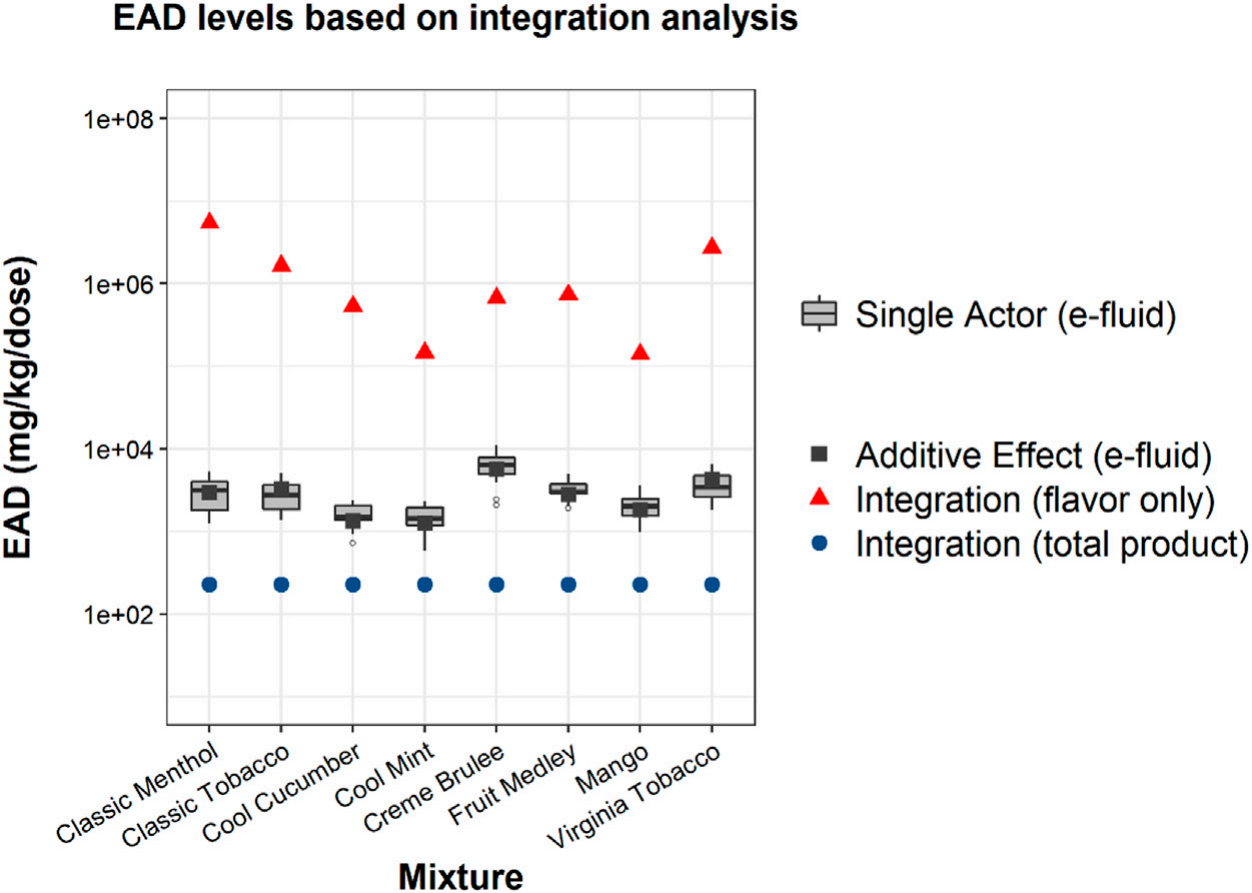
EAD predictions for each flavor using the ingredient integration analysis based on the geometric mean *in vitro* cytotoxicity assay AC50 from the HTS data for individual ingredients. This figure compares integration analysis results under different scenarios (red and blue points) to the e-liquid mixture results presented in Figure 1 (gray points and boxplots). Blue circular points show predictions for total product, while red triangle points show predictions for flavor ingredients only. Note that for this analysis ingredients that were inactive in cytotoxicity assays were excluded (Supplementary Table S3); nicotine, BA, and VG were among the inactive ingredients. The grey boxplots show the results of the single actor analysis for each e-liquid using the MTT assay for comparison, while the grey square points show the additive effect results.

Using the outcome-oriented ingredient integration approach, EAD predictions for the “total product” scenario (Figure 4, blue dots) were the lowest, with little difference observed across EC product flavors. This suggests that the EAD estimation was mainly determined by the large mass percentage of PG, a carrier ingredient with cytotoxicity demonstrated in the HTS assays. EAD predictions for the “flavor only” scenario (Figure 4 red triangle) were substantially higher than the “total product” scenario results and showed high variability across the eight EC aerosols. Full sets of EAD predictions for all the combinations of PBPK models and scenarios were provided in Supplementary Table S5. The single actor (Figure 4 grey boxplots) or additive effect e-liquid (Figure 4 grey squares) results derived from the mixture cytotoxicity data were added to compare with the “total product” results (Figure 4 blue dots) derived from the ingredient cytotoxicity data. The e-liquid results were approximately a factor of 10 higher than the “total product” results and showed good consistency between different approaches.

## Discussion

The Committee on Toxicity Testing and Assessment of Environmental Agents of the National Research Council (NRC, 2007) has envisioned toxicity testing is to transit from whole animal *in vivo* testing toward *in vitro* approaches conducted in human cells. Besides regular *in vitro* assays, a suite of HTS assays targeted to specific biochemical targets has been developed by Tox21 consortium (Dix et al., 2007; Tice et al., 2013) to aid this transition. At the same time, putting the *in vitro* test data into an *in vivo* context requires the IVIVE approach to extrapolate *in vitro* bioactivity to *in vivo* exposures and dosimetry. While conceptually feasible, there are many variables and assumptions before applying IVIVE, especially on mixtures such as EC aerosols, decisions such as what the expected bioactivity is, what components of the mixture contribute to the bioactivity, what *in vitro* assays will be used, and how that data will be integrated need to be determined as all of these influence the interpretation of model predictions. This work investigated these issues by using publicly available *in vitro* cytotoxicity data of EC aerosols and ingredients and comparing EADs estimated with various IVIVE data integration approaches. We evaluated the impact of e-liquid carriers (PG and VG) and BA on the total EC aerosol bioactivity and EAD predictions, albeit limited to available *in vitro* data for EC mixtures and ingredients. We also compared the EAD results from this study with results predicted by previous approaches (Chang et al., 2021).

### EAD Predictions for Total Product

Chang et al. (2021) estimated EADs of e-liquids using *in vitro* cytotoxicity and analytical data of EC mixtures reported in Omaiye et al. (2019). However, they did not account for PG, VG, and BA, of which the mass percentage in the e-liquids or aerosols were not available. With the composition information obtained from the FDA’s website (2020), we were able to calculate the EAD based on the total e-liquid composition (PG, VG, BA, nicotine, and flavors) using the EC aerosol MTT assay (Omaiye et al., 2019). As shown in Figure 1, the EAD predictions using both the single actor approach and the additive approach indicated that the inclusion of PG, VG or BA in IVIVE modeling did not have a significant impact on the human EADs based on the mixture MTT assay (Omaiye et al., 2019), despite that the three ingredients accounted for over 85% of the total mass of e-liquids and aerosols. The results can be explained by the distribution of the mixture bioactivity across all the ingredients and the relatively faster clearance of the carriers, which represent the majority of the mixture mass (Supplementary Table S1). The relatively high intrinsic clearance rate and fraction unbound of the carriers (Supplementary Table S4) contributed to their rapid clearance and resulted in a lower Cmax in the dosimetry model and thus a minor impact on the EAD.

This result based on total mixture (EC aerosols) *in vitro* bioactivity (Figure 1) seemed to contrast with the result of the single actor approach using individual ingredient *in vitro* HTS data. EAD estimations resulting from the HTS data of individual ingredients (Figure 2) indicated that the “total product” EAD might be lower than nicotine + flavors alone. Using the lowest AC50 value across multiple assays from the HTS data set, the ingredient-based single actor approach indicated that PG and BA consistently produced two of the three lowest EAD estimations immediately following nicotine (Figure 3). Using the lowest HTS AC50 among all assays is an extremely conservative approach and might have overestimated the bioactivity of these ingredients relative to what was observed in the *in vitro* mixture cytotoxicity testing. This was also partly due to the lack of bioactivity across the assays in general and the lowest measure is not related to cytotoxicity or generally not as robust due to experimental and technical variability. This discrepancy in the results highlights how assay selection and interpretation can impact IVIVE calculations.

Either the single actor or the additive effect approaches did not incorporate the biological mechanisms. When we used the single actor approach with the HTS data of individual ingredients, we selected the most sensitive assay among assays annotated with various mechanistic targets to obtain the most conservative EAD estimations. This has a disadvantage of being overly sensitive and may reflect a spurious interaction as opposed to a biologically relevant measure of ingredient effect on the biological system. Nonetheless, this preliminary approach can be used in screening and prioritizing when the goal is to be conservative to assess potential bioactivity. In contrast, when we used the additive effect approach with the mixture cytotoxicity data, we assumed equal toxic potential for all ingredients of the mixture, as individual ingredient bioactivity for the assay was not available. This assumption likely attributed bioactivity to relatively inert ingredients and could affect the EAD estimates when combined with different PK profiles.

### EAD Predictions Using an Outcome-Oriented Ingredient Integration Approach

As an alternative to the above approaches, we explored the mechanism-based IVIVE for the mixtures, an outcome-oriented ingredient integration approach using a common mechanism. This approach has the advantage of consolidating biological effects from ingredients that would contribute to the same toxicity endpoints in a mixture that has not yet been tested in experiments. In this regard, the outcome-oriented ingredient integration approach is similar to the toxicity equivalent factor (TEF) approach applied by the U.S. Environmental Protection Agency to assess the toxicity of structurally similar chemicals that affect the same endpoint (Delistraty, 1997). This approach also assumes additive effects from single ingredients. We selected cellular cytotoxicity to make relevant comparison to the results derived from the mixture cytotoxicity data (Omaiye et al., 2019).

The EAD estimations from the cytotoxicity-oriented ingredient integration approach encompassed a range of approximately 10-fold below to 100-fold above those estimated using the mixture cytotoxicity data, depending on which ingredients are included in the analysis (“total product” or “flavor only”) (Figure 4). Notably, the “total product” EAD estimation produced conservative results, i.e., the EAD predictions for the total product were the lowest. When comparing the outcome-oriented ingredient integration approach to the single actor approach based on the HTS ingredient data, the “total product” EAD estimations were the lowest (Figure 2; Figure 4). When comparing to the additive effect approach using mixture cytotoxicity data (Figure 4, grey points and boxplots), the results of the outcome-oriented ingredient integration approach are about a factor of 10 lower than the mixture cytotoxicity-based results, which could be explained in part by the inclusion of different ingredients and the varied sensitivity of the *in vitro* assays. Similar to the additive effect approach demonstrated in Figure 1 (also included in Figure 4 as gray square points), the outcome-oriented ingredient integration approach modeled the pharmacokinetics of each ingredient separately. The two approaches (the additive approach vs. the outcome-oriented ingredient integration approach) differ, however, in the weight of contribution that is assigned to each ingredient. The additive effect approach assumes that all ingredients contribute to the mixture cytotoxicity and every ingredient is equally cytotoxic, while the outcome-oriented ingredient integration approach assumes that only ingredients of which cytotoxicity is demonstrated (“positive” in Tox21/ToxCast database) and the contribution of a single ingredient is inversely proportional to its AC50 (Supplementary File).

Despite increased biological relevance, there are limitations of the outcome-oriented ingredient integration approach, for example, limited *in vitro* data relevant to mechanistic targets of interest. In this case study, there were only seven distinct ingredients with active *in vitro* responses out of the total 46 ingredients (Table 1). An additional 13 ingredients were considered active using the median AC50 of cytotoxicity assays (387.012 uM) for the calculation as a conservative estimation based on a lack of *in vitro* testing data. Nicotine and BA were excluded from the outcome-oriented integration modeling as they are both identified as inactive in the Tox21/ToxCast cell viability assays. Although cytotoxicity and cell viability assays were not available for all ingredients, it may be possible to address the data gap by searching the literature or conducting ingredient-specific *in vitro* experiments.

### EADs for Preliminary Risk Assessment

The methodologies presented in the work incorporated the bioactivity, ADME and composition of ingredients to enable preliminary chemical risk assessment. The results can be used to prioritize individual ingredients or ingredient groups for further toxicological testing and risk assessment. For EC products, flavors, nicotine (or nicotine salt), and humectant carriers can play different roles in the *in vitro* and *in vivo* responses and the IVIVE methods provided a modeling approach to estimate the potential contribution of each or groups of ingredients. For example using the HTS data, the EAD predictions for the “flavor only” group are the highest, suggesting flavors (less than 1.2% of the total mass in this case study) are not likely the major toxicity driver in the tested e-liquid and aerosol mixtures. The medians of “flavor-only” EAD predictions are about a factor of 100 higher than the “total product” results when the lowest AC50 data are used (Figure 2). The “flavor-only” predictions are about a factor of 10,000 higher than the “total product” results when the selected cytotoxicity data are used for the estimation (Figure 4). The difference between the “flavor only” and “total product” scenarios suggests the contribution of the flavor and non-flavor ingredients to the potential *in vivo* toxicity of the mixture. In addition, the IVIVE results can help to identify priority ingredients that may drive the bioactivity. Considering their bioactivity and levels, it is not surprising that nicotine and BA significantly contribute to the bioactivity of the mixture as shown in Figure 2. Among flavors, benzyl alcohol, identified as a flavor compound in six out of eight EC products, and ethyl maltol, identified in three products, could contribute to lower EADs among flavor ingredients (non-PG, VG, BA, or nicotine ingredients) across the EC products (Figure 3). Additional toxicological assessment (e.g., *in vitro* responses in various combinations of these ingredients) could be of interest to further elucidate their contributions to mixture bioactivity and to evaluate their use level in the products. For the EC aerosols as a whole, the lowest EAD predictions for the total product (>100 mg/ kg body weight, i.e. > 7,000 mg/ day based on 70-kg body weight, about ten pods per person per day) (Figure 4) were still regarded substantially higher than typical consumer daily uses (e.g., approximately two pods per person per day (0.7 ml of e-liquid per pod https://www.juul.com/resources/what-is-the-size-of-a-juulpod).

Despite many potential applications, it is important to acknowledge the limitations of the current IVIVE approaches for the EAD predictions. Firstly, although cytotoxicity was selected as the surrogate biological response in this study, *in vitro* assays that are more mechanistically relevant and specific to the exposure would be desirable for the EAD prediction and risk assessment. For example, increased vascular and lung oxidative stress level was reported to be associated with e-vapor aerosol exposure (Taylor et al., 2016; Kuntic et al., 2019) and may lead to potential lung injury. Secondly, the *in vitro* values used did not account for any potential interaction with endogenous ligands for the targeted receptors. Additionally, the metabolic saturation was not considered in the generic PBPK model used in this study. In future studies, the PBPK model may be expanded by adding key metabolic information (e.g. saturable metabolic pathways and resulting metabolites). Target tissue concentrations can also be considered for the EAD calculation in addition to the systemic plasma concentration. Finally, for EAD calculation of mixtures, we used the simple assumptions on the interaction of ingredients (additive as opposed to synergistic, for instance). For a simple mixture system such as a binary system, the interactions between chemicals could possibly be incorporated in the model and verified by experiments. Considering these limitations and data gap, uncertainty factors can also be applied to EAD calculation depending on the application (Raies and Bajic, 2016).

## Conclusion

IVIVE modeling for mixtures can be complex as it must consider both the bioactivity measure and the pharmacokinetics of each ingredient in the mixture. This work investigated the application of IVIVE as a NAM to use *in vitro* data toxicity assessment of EC ingredients separately and as part of total product in the context of human *in vivo* exposure. Specifically, we illustrated the impact of the inclusion or exclusion of carrier chemicals (PG and VG) and BA in e-liquids and EC aerosols on EAD prediction using various IVIVE approaches. The single actor analysis demonstrated in this work can be informative for prioritization of testing for ingredients or in sets of mixtures. By considering bioactivity, mass fraction, and toxicokinetic properties, the IVIVE results can identify which ingredients in a set of mixtures are most likely to contribute to toxic effects, potentially supporting usage limit (Figure 3). We also demonstrated that the outcome-oriented ingredient integration approach as introduced in this work can provide conservative screening estimates of mixture bioactivity for selected bioactivity targets when experimental mixture data are not available. The EAD estimations generated by these approaches have the potential to inform risk assessment and decision making through a margin of exposure approach that compares expected exposures with the equivalent exposures to match *in vitro* results. As different assumptions are embedded in each approach, it is necessary to define the purpose of the study and the assumptions to develop fit-for-purpose methodologies. In conclusion, IVIVE is a useful tool for interpreting *in vitro* data in the context of *in vivo* human exposure and can be applied to mixtures assessment for hypothesis generation and preliminary risk assessment.

## Data Availability

The original contributions presented in the study are included in the article/Supplementary Material, further inquiries can be directed to the corresponding author.
