# Supplementary material for: Evaluation of Inhalation Exposures and Potential Health Impacts of Ingredient Mixtures Using in vitro to in vivo Extrapolation
Source: Front Toxicol. 2022 Feb 2;3:787756. doi: 10.3389/ftox.2021.787756 (PMC8915826; doi:10.3389/ftox.2021.787756)
Supplement: Supplementary file 1 [file Presentation1.zip › Supplementary Material_Dec21 2021/Altria_014_TotalProduct_Supplemental File_20210520.docx]

**Supplemental Materials for “Evaluation of exposure and health impacts of mixture ingredients using in vitro to in vivo extrapolation”**

Jingjia Zhang^1^*, Tessa Holland^2^, Xiaoqing Chang^3^, David E. Hines^3^, Agnes L. Karmaus^3^, Shannon Bell^3^, and K. Monica Lee^1^

^1^Altria Client Services, LLC, 6603 W Broad St, Richmond, VA 23230, USA

^2^Lancaster Laboratories, c/o Altria Client Services, Research, Development & Regulatory Affairs, Richmond, VA

^3^Integrated Laboratory Systems, LLC, 601 Keystone Park Drive, Suite 200, Morrisville, NC 27560, USA

**Example ingredient integration analysis calculations.** Here, we demonstrate the ingredient integration analysis calculations using a simplified example case study that considered a mixture of three hypothetical chemicals (“A”, “B”, and “C”) in an inert solvent. The input data for the analysis are provided in Table 1, which shows mixture composition (% of total mixture), bioactivity data for the individual ingredients (hypothetical active concentration 50% [AC50], represented in mg/L for ease of calculation), the maximum plasma concentration (Cmax) of each ingredient resulting from a single 1mg/kg dose of the mixture (Cmax_mix_), and the chemical-specific pharmacokinetic (PK) parameters used to calculate Cmax values. The PK parameters for each chemical were obtained from the internal parameter database included in the httk R package (Wambaugh et al., 2016). Specifically, we used the parameters for CAS numbers 50-49-7, 51384-51-1, and 15972-60-8 as data sources for the hypothetical chemicals A, B, and C, respectively. These data were used to parameterize the “solve_3comp” model option in the httk package, which contains compartments for gut, liver, and the rest of the body (R. G. Pearce et al., 2017). Default values were used unless otherwise stated.

Table 1 Input data for hypothetical mixture used in ingredient-based integration example calculation

| Parameters | Description | Chemical A | Chemical B | Chemical C |
| --- | --- | --- | --- | --- |
| % w/w of Mixture | % ingredient composition by mass | 10 | 4 | 2 |
| AC50 (mg/L)^†^ | Hypothetical AC50 | 4 | 20 | 0.2 |
| Cmax_mix_i_ (mg/L)^‡^ | Cmax predicted from 1 mg/kg mixture exposure | 0.1269 | 0.0507 | 0.0254 |
| Clint | intrinsic clearance (ul/min/10^6^ cells) | 6.308 | 5.607 | 62.9 |
| Fgutabs | Fraction of the oral dose absorbed | 1 | 1 | 1 |
| Fhep.assay.correction | Fraction unbound in hepatocyte assay | 0.2109 | 0.8934 | 0.667 |
| Funbound.plasma | Fraction of plasma that is not bound | 0.1 | 0.883 | 0.133 |
| Hematocrit | Percent volume of red blood cells in blood | 0.44 | 0.44 | 0.44 |
| Kgut2pu | Gut to unbound plasma concentration ratio | 1904 | 6.412 | 18.02 |
| Kgutabs | Lumen to gut uptake rate (h^-1^) | 2.18 | 2.18 | 2.18 |
| Kliver2pu | Liver to unbound plasma concentration ratio | 3112 | 11.3 | 167.2 |
| Krbc2pu | Red blood cell to unbound plasma concentration ratio | 110.8 | 1.698 | 7.236 |
| Krest2pu | Rest of body to unbound plasma concentration ratio | 1020 | 3.604 | 54.15 |

† Hypothetical AC50 values; ‡ Cmax of the ingredient based on 1mg/kg exposure of the mixture as calculated in Table 2.

After defining the mixture components and composition, individual Cmax values are calculated for a 1 mg/kg dose of each ingredient (Cmax_1_). The Cmax_1_ values for each chemical used in the example calculation are the same due to the simplified nature of the 3 compartment httk model and the IV exposure route, but it should be noted that this is not true for all chemicals. These Cmax_1_ values are then multiplied by the mass percent composition of the ingredient in the mixture to calculate the Cmax­_mix_ as is shown in Table 2.

Next, the AC50 values of individual chemicals are used to determine the relative activity of each constituent. Specifically, the ratios of AC50 values are used to describe the relative potency of each chemical and to express the predicted Cmaxmix_i values from each chemical in units of a single constituent. While any of the chemicals in a mixture can be used for this analysis and all will provide computationally identical results, we chose chemical C because it had the lowest AC50 value (0.2 mg/L). Thus, the potency of each ingredient (p_­i_) was calculated as $p_{i}=\frac{{AC50}_{i}}{{AC50}_{C}}$.

The Cmax_mix_i_ of each chemical can then be divided by the relative potency of each chemical (pi) to obtain the relative Cmax of that component in units of chemical C (${Relative Cmax}_{i}=\frac{{Cmax}_{i}}{p_{i}}$), as can be seen in Table 2. In this example, chemical C is 20 times more potent than chemical A, thus the chemical A Cmax that is predicted to result from a 1 mg/kg exposure to the mixture (Cmax_mix_A_ = 0.1268 mg/L) would be expected to have the same effect as a Cmax for chemical C of 0.0063 mg/L. The relative Cmax values for each ingredient can then be summed to obtain the total Cmax of the mixture (mixture effective Cmax) because these values are all in units of chemical C (Table 2).

Table 2 Example calculation of ingredient relative Cmax and mixture effective Cmax for hypothetical mixture

| Chemical | Cmax_1_ (mg/L) | | % w/w of mixture | | Cmax_mix_ (mg/L) | Potency  (*p*) | Relative Cmax (mgC/L) |
| --- | --- | --- | --- | --- | --- | --- | --- |
| A | 1.268 | | 10 | | 0.1268 | 20 | 0.0063 |
| B | 1.268 | | 4 | | 0.0507 | 100 | 0.0005 |
| C | 1.268 | | 2 | | 0.0254 | 1 | 0.0254 |
|  | |  | | Mixture effective Cmax: | | | 0.0322 |

Finally, the mixture effective Cmax can be used in conjunction with the AC50 for the most sensitive ingredient to estimate the EAD of the mixture that would be necessary to achieve 50% activity as $EAD=\frac{{AC50}_{most sensitive}}{Mixture effective Cmax}$ . In the example calculation, chemical C had the lowest AC50 (0.2 mg/L), thus we used this AC50 to compute an EAD of 6.02 mg/kg for the hypothetical mixture.

**References**

Pearce, R., Strope, C., Setzer, W., Sipes, N., Wambaugh, J., 2017. httk: R Package for High-Throughput Toxicokinetics. Journal of Statistical Software 79. https://doi.org/10.18637/jss.v079.i04

Wambaugh, J., Pearce, R., Davis, J., Setzer, R.W., Sipes, N., 2016. httk: High-Throughput Toxicokinetics. R package version 1.4.6
