# Supplementary material for: Evaluation of Inhalation Exposures and Potential Health Impacts of Ingredient Mixtures Using in vitro to in vivo Extrapolation
Source: Front Toxicol. 2022 Feb 2;3:787756. doi: 10.3389/ftox.2021.787756 (PMC8915826; doi:10.3389/ftox.2021.787756)
Supplement: Supplementary file 1 [file Presentation1.zip › Supplementary Material_Dec21 2021/Manuscript_Supplemental_File 1.pdf]

# R Notebook

Code ▾

## Outcome-orientated integration approach


## Author: David Hines (dhines@ils-inc.com (mailto:dhines@ils-inc.com))



## License: MIT


## Version: 1.0

## Supplemental example code for the manuscript

## “Evaluation of inhalation exposures and potential health impacts of ingredient mixtures using in vitro to in vivo extrapolation”


## Authors:

## Jingjie Zhang, Xiaoqing Chang, Tessa Holland, David E. Hines, Agnes L. Karmaus, Shannon Bell, and K. Monica Lee


## November 23, 2021

This notebook is meant to present an example application of the methodology for the outcome-oriented integration of ingredient-level bioactivity when multiple constituents in a mixture affect the same toxicity endpoint. This methodology will be presented using a hypothetical scenario.

Parmacokinetic (PK) parameters for this hypothetical example will be obtained from the default database that is included in the httk package. The three compounds that will be included in the hypothetical mixture are:

```
COMPOUND DATA   | LABEL |        CASN
======================================
Imipramine      |   A   |     50-49-7
Metoprolol      |   B   |  51384-51-1
Alachlor        |   C   |  15972-60-8
```

These compounds were selected because complete parameter sets (PK parameters, clearance parameters, fraction unbound in plasma) are available in the httk package. This allows us to focus on the mathematical approach without spending time focusing on gathering data. The compounds have been given new labels (A, B, and C) to emphasize that we are using these compounds as part of a methods presentation, and that the results are not relevant for these substances. Additionally, these compounds do not act on the same toxicity endpoint in reality, so we'll introduce a hypothetical assay with an endpoint that is affected by all three compounds.

We will assume that we have an assay that indicates activation of a toxicity pathway through inhibition of the binding of an ion to a pump. This will be called "Hypothetical Binding Inhibition" (HBI). Hypothetical assay data with respect to HBI will be provided to inform the bioactivity of each compound.

The workflow for this notebook will be as follows:

1. Load required packages
2. Define mixture composition
3. Create hypothetical in vitro assay data for HBI
4. Calculate PK profiles for each compound
5. Calculate EAD predictions

#—————— # 1) Load packages #——————#

Load required packages

Hide

```
library(plyr)
library(dplyr)
library(deSolve)
library(httk)
library(tidyr)  #example function:gather()
library(ggplot2) #example function:ggplot()
library(drc)
```

#—————— # 2) Define mixture composition #——————#

Now we need to provide the CAS numbers for the three chemicals in our hypothetical mixture

Hide

```
cas.mix <- c('50-49-7', '51384-51-1', '15972-60-8')
names(cas.mix) <- c('A','B','C')
```

We also need to know how much of each chemical is in the mixture. Notice that the percentages don't add up to 100%. This is because the percentage given is as a total of the administered mixture, and these active chemicals are only a portion of a larger hypothetical mixture.

Hide

```
chemical <- c('A','B','C')
MW <- chem.invivo.PK.aggregate.data$MW[
  which(chem.invivo.PK.aggregate.data$CAS %in% cas.mix)]
concentration <- c(0.35, 0.06, 0.025) # percent of total mixture

mix <- data.frame(chemical, cas.mix, MW, concentration)
colnames(mix) <- c('Chemical','CAS','MW','Percent')
mix
```

| | Chemical <chr> | CAS <chr> | MW <dbl> | Percent <dbl> |
|---|---|---|---|---|
| A | A | 50-49-7 | 269.770 | 0.350 |
| B | B | 51384-51-1 | 280.415 | 0.060 |
| C | C | 15972-60-8 | 267.369 | 0.025 |

3 rows

#———- # 3) Create hypothetical in vitro assay data for HBI #———-#

We need to know about how each chemical in the mixture affects our hypothetical binding inhibition (HBI) assay. We will first introduce assay data for each chemical, which will be used to calculate an IC50 for each chemical. The HBI-assay data for each chemical are as follows:

Hide

```
# -------- chemical A --------
#create hypothetical assay data
binding.A <- c(100.0,96.8,99.4, # % binding vs control
               98.9,99.5,97.9,
               64.5,76.7,70.2,
               33.2,29.1,40.4,
               20.1,24.2,13.1,
               1.1,0.6,0.1,
               0.2,0.0,0.1)
conc.A <- c(rep(1e-1, 3), # unit is ng/mL
            rep(1e-0, 3),
            rep(1e+1, 3),
            rep(1e+2, 3),
            rep(1e+3, 3),
            rep(1e+4, 3),
            rep(1e+5, 3))

chem.A_raw_HBI <- data.frame(binding.A, conc.A)

# -------- chemical B --------
#create hypothetical assay data
binding.B <- c(100.0,96.8,99.4, # % binding vs control
               98.9,99.5,97.9,
               95.1,86.7,90.2,
               89.9,81.2,74.2,
               30.2,20.1,21.5,
               0.1,4.2,1.1,
               0.3,0.1,0.1)
conc.B <- c(rep(1e-1, 3), # unit is ng/mL
            rep(1e-0, 3),
            rep(1e+1, 3),
            rep(1e+2, 3),
            rep(1e+3, 3),
            rep(1e+4, 3),
            rep(1e+5, 3))

chem.B_raw_HBI <- data.frame(binding.B, conc.B)

# -------- chemical C --------
#create hypothetical assay data
binding.C <- c(100.0,96.8,99.4, # % binding vs control
               74.9,69.5,62.9,
               30.5,27.7,19.2,
               13.2,5.1,1.4,
               0.1,0.2,1.1,
               0.2,0.1,0.0,
               0.1,0.0,0.0)
conc.C <- c(rep(1e-1, 3), # unit is ng/mL
            rep(1e-0, 3),
            rep(1e+1, 3),
            rep(1e+2, 3),
            rep(1e+3, 3),
            rep(1e+4, 3),
```

```
            rep(1e+5, 3))

chem.C_raw_HBI <- data.frame(binding.C, conc.C)
```

Next, we'll use the drm function in the drc package to create a dose-response model. We'll then estimate an IC50 for each chemical using the ED function. We've also included a plot for each chemical to visualize the results

Hide

```
# -------- chemical A --------
#construct a dose-response model with the drc package
dr_A <- drm(binding.A ~ conc.A, data=chem.A_raw_HBI, fct = LL.4())
```

```
NaNs producedNaNs producedNaNs producedNaNs producedNaNs producedNaNs produced
```

Hide

```
#plot just to visualize
newdata <- expand.grid(conc=exp(seq(log(1e-1), log(1e+5), length=1000)))
pred.A <- predict(dr_A, newdata=newdata)

#calcuate IC50
ed.A <- cbind(newdata, pred.A)
ic50.A <- ed.A$conc[which.min(abs(ed.A$pred.A-50))]

par(las=1)
plot(chem.A_raw_HBI$conc.A, chem.A_raw_HBI$binding.A,
     log='x',
     xlab=('Chemical A Concentration (ng/mL)'),
     ylab='HBI binding results (% control)')
lines(x=unlist(newdata), y=pred.A)
```

Hide

```
lines(x=c(ic50.A[1], ic50.A[1]), y=c(-100,1000), lty=2, col='red')
lines(x=c(1e-6,1e6), y=c(50,50), lty=2, col='gray')
```

Hide

```
# -------- chemical B --------
#construct a dose-response model with the drc package
dr_B <- drm(binding.B ~ conc.B, data=chem.B_raw_HBI, fct = LL.4())

#plot just to visualize
pred.B <- predict(dr_B, newdata=newdata)

#calcuate IC50
ed.B <- cbind(newdata, pred.B)
ic50.B <- ed.B$conc[which.min(abs(ed.B$pred.B-50))]

par(las=1)
```

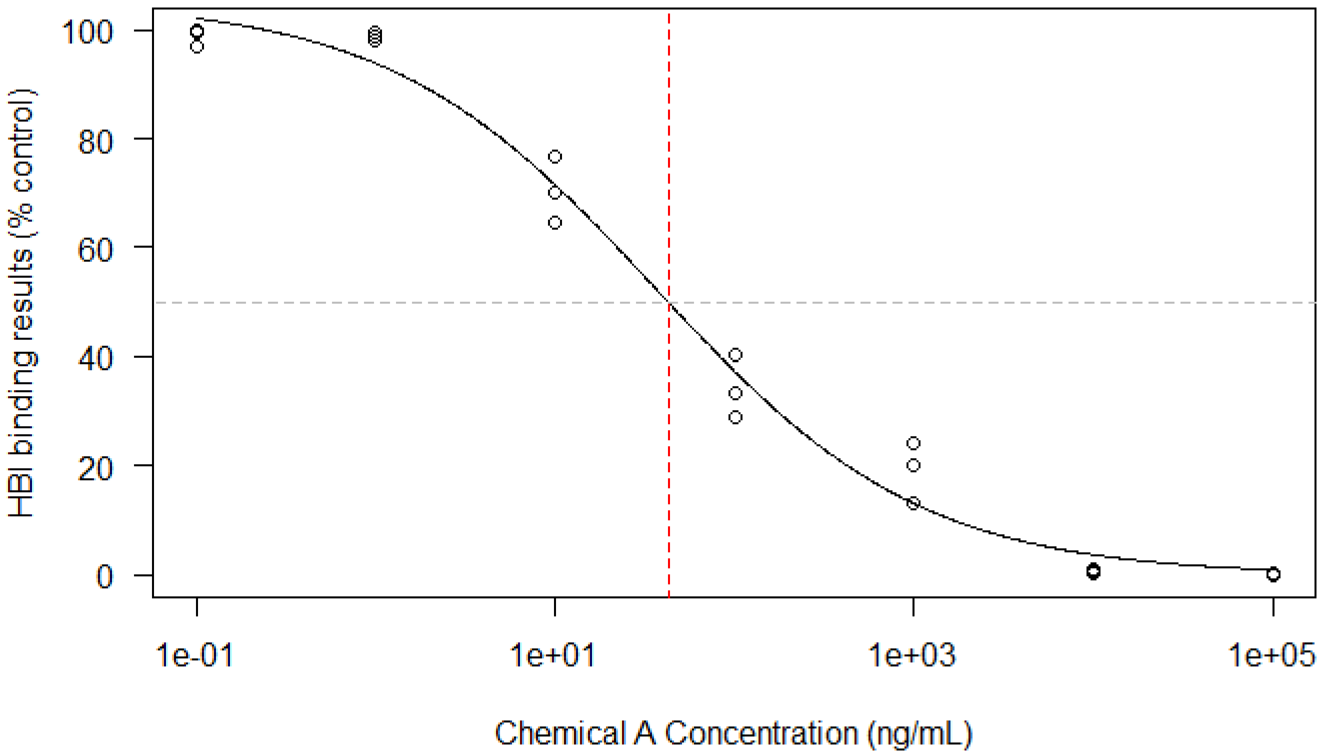


Hide

```
plot(chem.B_raw_HBI$conc.B, chem.B_raw_HBI$binding.B,
     log='x',
     xlab=('Chemical B Concentration (ng/mL)'),
     ylab='HBI binding results (% control)')
lines(x=unlist(newdata), y=pred.B)
```

Hide

```
lines(x=c(ic50.B[1], ic50.B[1]), y=c(-100,1000), lty=2, col='red')
lines(x=c(1e-6,1e6), y=c(50,50), lty=2, col='gray')
```

Hide

```
# -------- chemical C --------
#construct a dose-response model with the drc package
dr_C <- drm(binding.C ~ conc.C, data=chem.C_raw_HBI, fct = LL.4())
```

```
NaNs producedNaNs producedNaNs producedNaNs producedNaNs producedNaNs producedNaNs producedNaNs
producedNaNs producedNaNs producedNaNs producedNaNs producedNaNs producedNaNs producedNaNs produ
cedNaNs producedNaNs producedNaNs producedNaNs producedNaNs produced
```

Hide

```
#plot just to visualize
pred.C <- predict(dr_C, newdata=newdata)

#calcuate IC50
ed.C <- cbind(newdata, pred.C)
ic50.C <- ed.C$conc[which.min(abs(ed.C$pred.C-50))]

par(las=1)
```

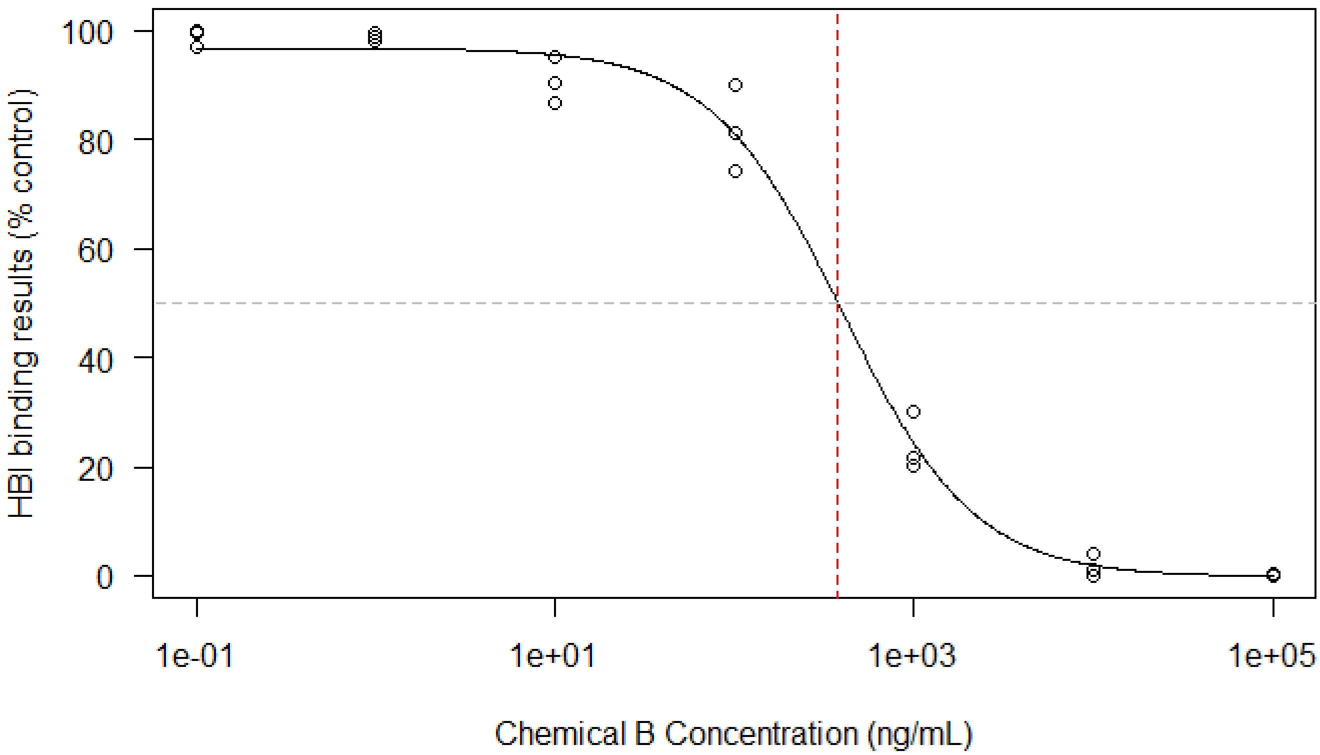


Hide

```
plot(chem.C_raw_HBI$conc.C, chem.C_raw_HBI$binding.C,
     log='x',
     xlab=('Chemical C Concentration (ng/mL)'),
     ylab='HBI binding results (% control)')
lines(x=unlist(newdata), y=pred.C)
```

Hide

```
lines(x=c(ic50.C[1], ic50.C[1]), y=c(-100,1000), lty=2, col='red')
lines(x=c(1e-6,1e6), y=c(50,50), lty=2, col='gray')
```

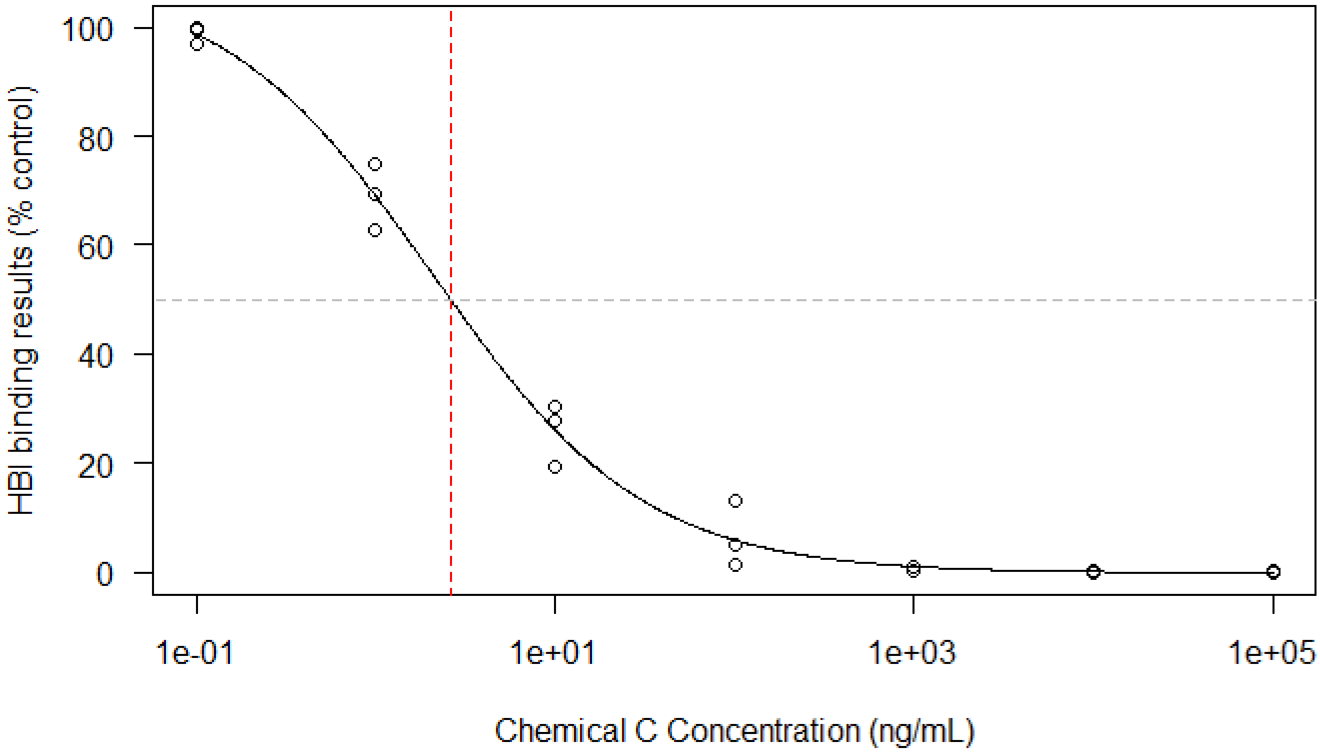


The IC50s for our three chemicals in the HBI detection assay are as follows. The units here are ng/mL to make the calculations/interpretations as simple as possible.

Hide

```
#report as a table
cat(paste(
'              |   IC50  \n',
'================================\n',
'Chemical A  |  ',formatC(ic50.A, format='e', digits=2),'ng/mL\n',
'Chemical B  |  ',formatC(ic50.B, format='e', digits=2),'ng/mL\n',
'Chemical C  |  ',formatC(ic50.C, format='e', digits=2),'ng/mL'))
```

```
             |   IC50
================================
Chemical A  |   4.27e+01 ng/mL
Chemical B  |   3.75e+02 ng/mL
Chemical C  |   2.61e+00 ng/mL
```

#—— # 4) Calculate PK profiles for each compound #——#

Now we need to calculate PK profiles for each compound. We will assume that the HBI pathway is initiated in the blood, and so we will focus on plasma concentration for each compound. We will use the solve_pbtk model from the httk package and assume an oral exposure route. The first thing we need to do is define the model run conditions:

Hide

```
#model selection conditions
species <- "human"              #"human" or "rat"
route="oral"                    #exposure route
modelType= "Solve_pbtk"         #Solve_3comp", "Solve_pbtk", or "solve_gas_pbtk"

#model dosing conditions
interv <- 24                    #assume 24hrs between doses
dpd <- 24/interv                #doses per day
inputDose_A <- 1                #mg/kg/d
inputDose_B <- 1                #mg/kg/d
inputDose_C <- 1                #mg/kg/d
ndays <- 1                      #days of dosing and simulation

ConcentrationUnit <- "mg/L"     #"mg/L"
```

Next we need to create parameter objects for each of the 3 chemicals in the mixture

Hide

```
# -------- chemical A --------
param.A_pbtk <- parameterize_pbtk(chem.cas = cas.mix[1],
                                  species = species,
                                  adjusted.Funbound.plasma = FALSE)
```

```
Human in vivo measured Rblood2plasma used.
```

Hide

```
# -------- chemical B --------
param.B_pbtk <- parameterize_pbtk(chem.cas = cas.mix[2],
                                  species = species,
                                  adjusted.Funbound.plasma = FALSE)
```

```
Membrane affintity (MA) predicted with method of Yun and Edginton (2013)Human in vivo measured R
blood2plasma used.
```

Hide

```
# -------- chemical C --------
param.C_pbtk <- parameterize_pbtk(chem.cas = cas.mix[3],
                                  species = species,
                                  adjusted.Funbound.plasma = FALSE)
```

```
Membrane affintity (MA) predicted with method of Yun and Edginton (2013)Human in vivo measured R
blood2plasma used.
```

Now can solve the model for each of the different chemicals

Hide

```
# -------- chemical A --------
#solve model equations
outA.0 <- solve_pbtk(chem.cas = cas.mix[1],
                     parameters = param.A_pbtk,
                     doses.per.day = dpd,
                     days = ndays,
                     tsteps = 12,
                     dose = 0,
                     daily.dose = inputDose_A*dpd,
                     iv.dose = FALSE, #note, FALSE because this is oral
                     output.units = ConcentrationUnit,
                     species = species,
                     default.to.human = TRUE,
                     plots = F,
                     suppress.messages = TRUE)

#store output
outA <- as.data.frame(outA.0)[,c(1:10,13)]

#convert units and store output
time.h <- sapply(outA[,1], function(x) x*24) #days to hours

outA.ngmL <- sapply(outA[,3:11], function(x) x*1000) #mg/L to ng/mL

out.A_1 <- as.data.frame(cbind(time.h, outA.ngmL)) # unit is ng/mL

# -------- chemical B --------
#solve model equations
outB.0 <- solve_pbtk(chem.cas = cas.mix[2],
                     parameters = param.B_pbtk,
                     doses.per.day = dpd,
                     days = ndays,
                     tsteps = 12,
                     dose = 0,
                     daily.dose = inputDose_B*dpd,
                     iv.dose = FALSE, #note, FALSE because this is oral
                     output.units = ConcentrationUnit,
                     species = species,
                     default.to.human = TRUE,
                     plots = F,
                     suppress.messages = TRUE)

#store output
outB <- as.data.frame(outB.0)[,c(1:10,13)]

#convert units and store output
time.h <- sapply(outB[,1], function(x) x*24) #days to hours

outB.ngmL <- sapply(outB[,3:11], function(x) x*1000) #mg/L to ng/mL

out.B_1 <- as.data.frame(cbind(time.h, outB.ngmL)) # unit is ng/mL

# -------- chemical C --------
```

```
#solve model equations
outC.0 <- solve_pbtk(chem.cas = cas.mix[3],
                     parameters = param.C_pbtk,
                     doses.per.day = dpd,
                     days = ndays,
                     tsteps = 12,
                     dose = 0,
                     daily.dose = inputDose_C*dpd,
                     iv.dose = FALSE, #note, FALSE because this is oral
                     output.units = ConcentrationUnit,
                     species = species,
                     default.to.human = TRUE,
                     plots = F,
                     suppress.messages = TRUE)

#store output
outC <- as.data.frame(outC.0)[,c(1:10,13)]

#convert units and store output
time.h <- sapply(outC[,1], function(x) x*24) #days to hours

outC.ngmL <- sapply(outC[,3:11], function(x) x*1000) #mg/L to ng/mL

out.C_1 <- as.data.frame(cbind(time.h, outC.ngmL)) # unit is ng/mL
```

Next, well have a quick look at what the different PK profiles look like for each one of our chemicals with a dose of 1 mg/kg

Hide

```
#indivisual plots
par(las=1)
plot(out.A_1$time.h, out.A_1$Cplasma,
     xlab='Time (hours)', ylab='Plasma Conc. A (ng/mL)')
```

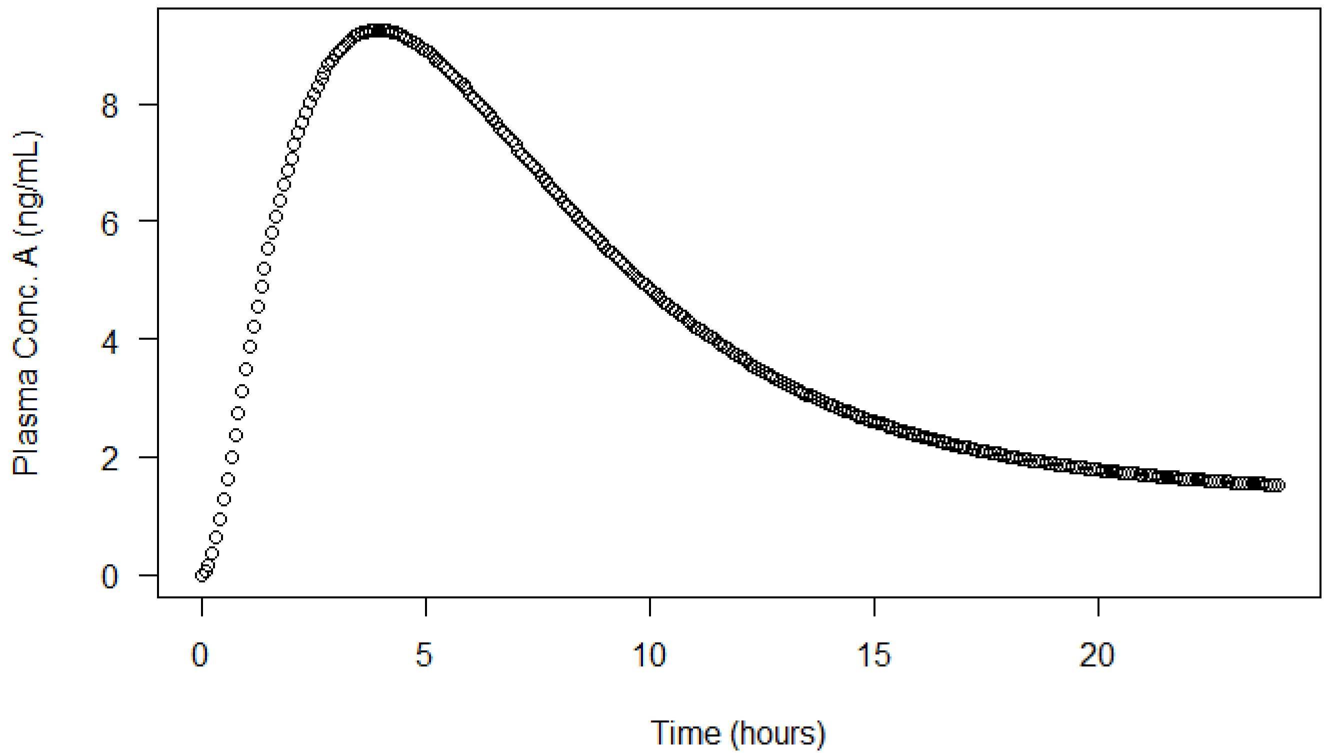


Hide

```
plot(out.B_1$time.h, out.B_1$Cplasma,
     xlab='Time (hours)', ylab='Plasma Conc. B (ng/mL)')
```

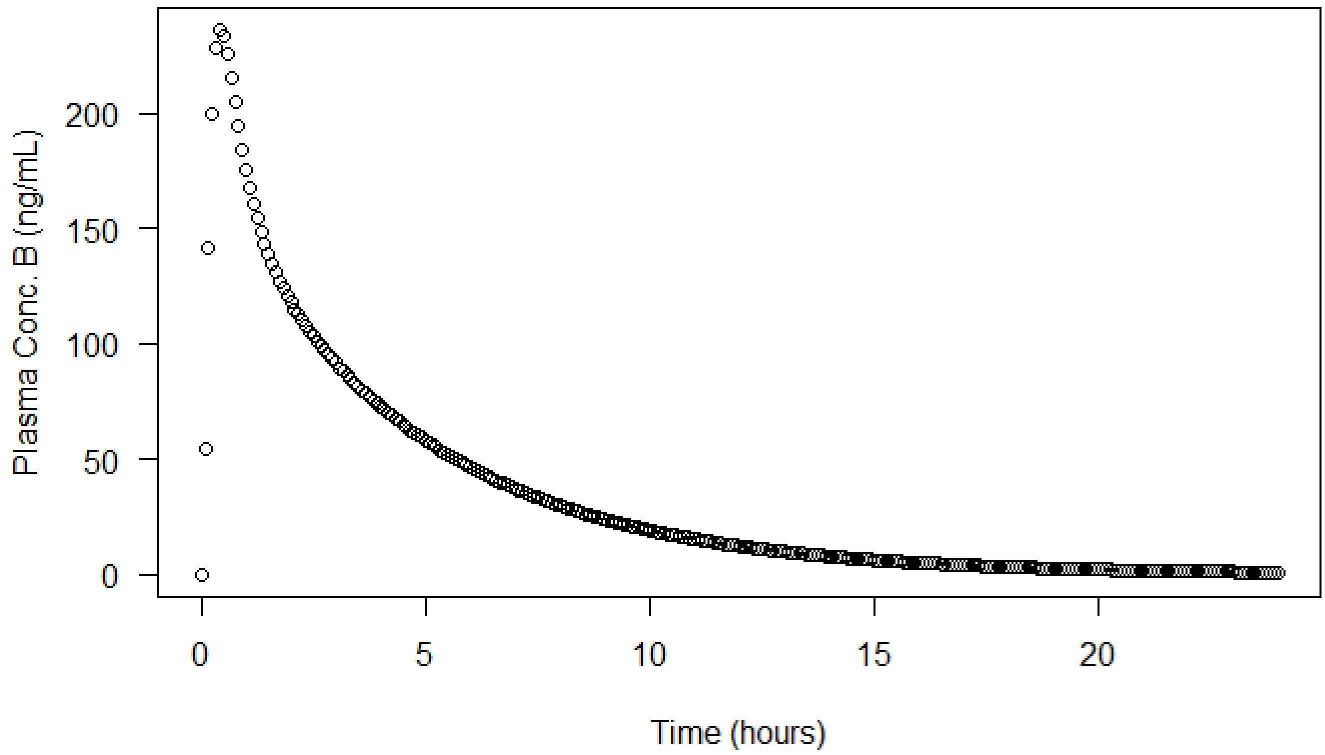

Hide

```
plot(out.C_1$time.h, out.C_1$Cplasma,
     xlab='Time (hours)', ylab='Plasma Conc. C (ng/mL)')
```

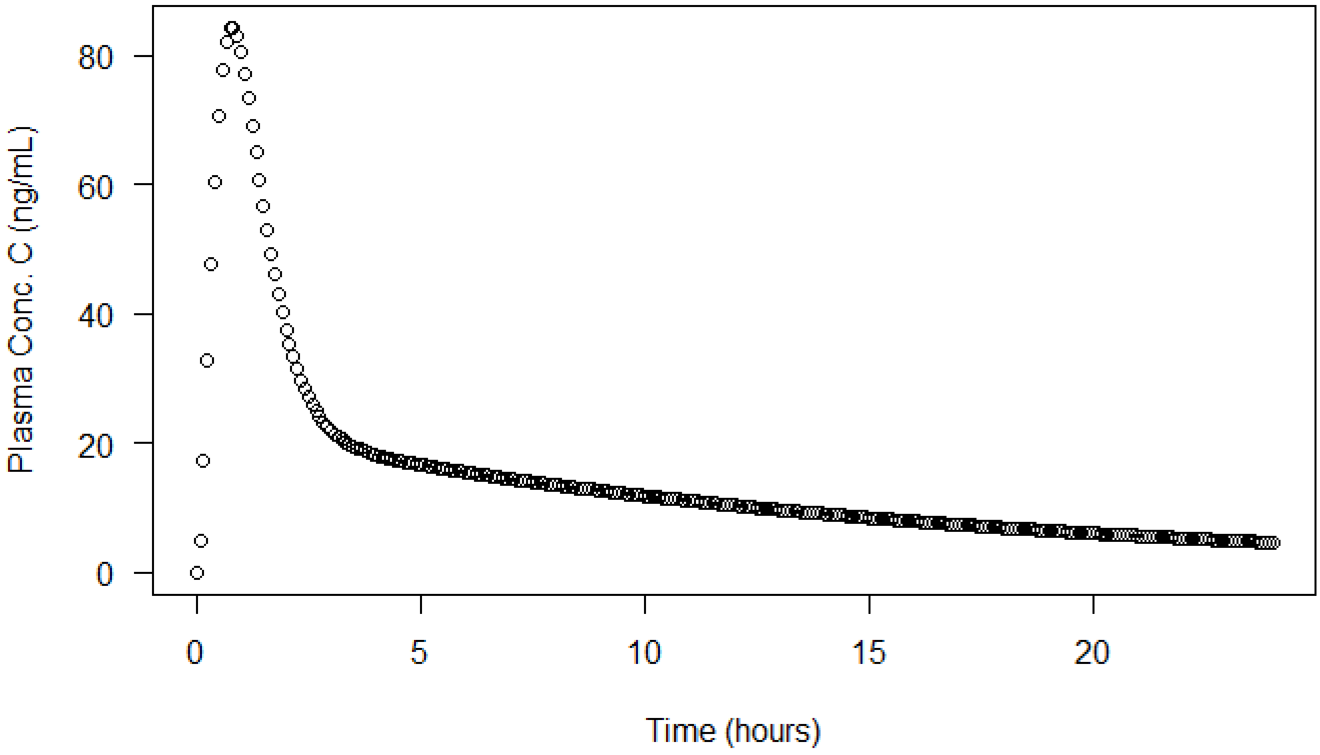


Hide

```
#all on one set of axes
plot(out.A_1$time.h, out.A_1$Cplasma,
     xlab='Time (hours)', ylab='Plasma Conc. (ng/mL)',
     ylim=c(0,250), type='l',
     col='black', lwd=2)
lines(out.B_1$time.h, out.B_1$Cplasma,
     col='red', lwd=2)
```

Hide

```
lines(out.C_1$time.h, out.C_1$Cplasma,
     col='dodgerblue', lwd=2)
legend('topright', legend=c('A','B','C'),
       lty=1, lwd=2,bty='n',
       col=c('black','red','dodgerblue'))
```

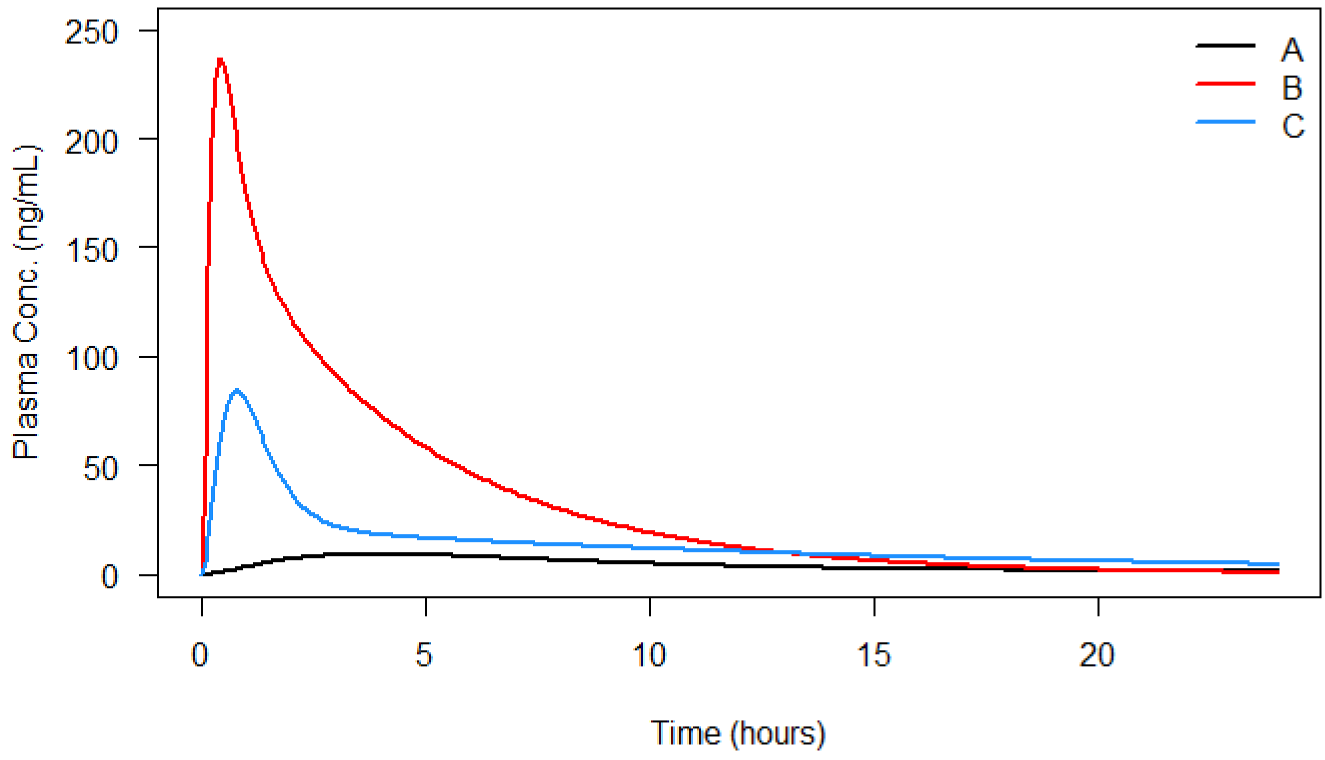


#——————— # 5) Calculate EAD predictions #——————#

Now that we have defined a mixture and bioactivity, we can calculate an EAD for the mixture. First, the Cmax for each ingredient in a mixture is converted to "relative units" of the most sensitive ingredient. Then, the relative Cmax values are summed and the resulting total Cmax is compared to the IC50 of the most sensitive ingredient to predict an EAD for the mixture.

This approach assumes 1) all effects are additive, 2) all ingredients reach Cmax at the same time, and 3) the relative effect of a chemical does not change with dose.

From section 3, we see that Chemical C has the lowest IC50 value at 2.61 ng/mL

The IC50 values for the three chemicals in the mixture were:

Chemical A | 4.27+01 ng/mL Chemical B | 3.74e+02 ng/mL Chemical C | 2.61e+00 ng/mL

For the simple integration approach, we need the we potency of each chemical relative to chemical C, which we can get by looking at the reciprocal of the ratio of the IC50s:

Hide

```
#calculate relative bioactivity
potency_A <- 1/(ic50.A/ic50.C)
potency_B <- 1/(ic50.B/ic50.C)
potency_C <- 1/(ic50.C/ic50.C)

#report as a table
cat(paste(
'              |   Activity \n',
'============================\n',
'Chemical A  |  ',round(potency_A, digits=4),'\n',
'Chemical B  |  ',round(potency_B, digits=4),'\n',
'Chemical C  |  ',round(potency_C, digits=4)))
```

```
             |   Activity
 ============================
 Chemical A  |   0.0612
 Chemical B  |   0.007
 Chemical C  |   1
```

Next, the Cmax values from 1mg/kg exposures for each ingredient (predicted in section 4) are scaled by the potency factors and mixture composition percentage to calculate the effective Cmax of that chemical in the mixture (relative to chemical C). These are then summed to get the total effective Cmax of the mixture.

Hide

```
#ingredient Cmax (telative to chemical C)
mix_cmax_A <- max(out.A_1$Cplasma)*potency_A * mix$Percent[1]
mix_cmax_B <- max(out.B_1$Cplasma)*potency_B * mix$Percent[2]
mix_cmax_C <- max(out.C_1$Cplasma)*potency_C * mix$Percent[3]

#mixture effective Cmax
effective_cmax <- sum(c(mix_cmax_A, mix_cmax_B, mix_cmax_C))
```

Finally, we calculate EAD using reverse dosimetry and linear extrapolation. Specifically, we look at the IC50 of chemical C relative to the mixture effective Cmax.

Hide

```
EAD <- ic50.C/effective_cmax
cat(paste('Outcome-oriented integration EAD =', round(EAD, digits=4), 'mg/kg'))
```

```
Outcome-oriented integration EAD = 1.0874 mg/kg
```
