# Supplementary material for: Evaluation of Inhalation Exposures and Potential Health Impacts of Ingredient Mixtures Using in vitro to in vivo Extrapolation
Source: Front Toxicol. 2022 Feb 2;3:787756. doi: 10.3389/ftox.2021.787756 (PMC8915826; doi:10.3389/ftox.2021.787756)
Supplement: Supplementary file 1 [file Presentation1.zip › Supplementary Material_Dec21 2021/Supplemental Figures_20211222.pdf]

# Supplemental figures

Supp. Figure S1A. EAD and pod comparison between inclusion and exclusion of carriers and BA, MTT assay, 24-hr and 2-hr interval , Gas_PBTK model

Figure S1A

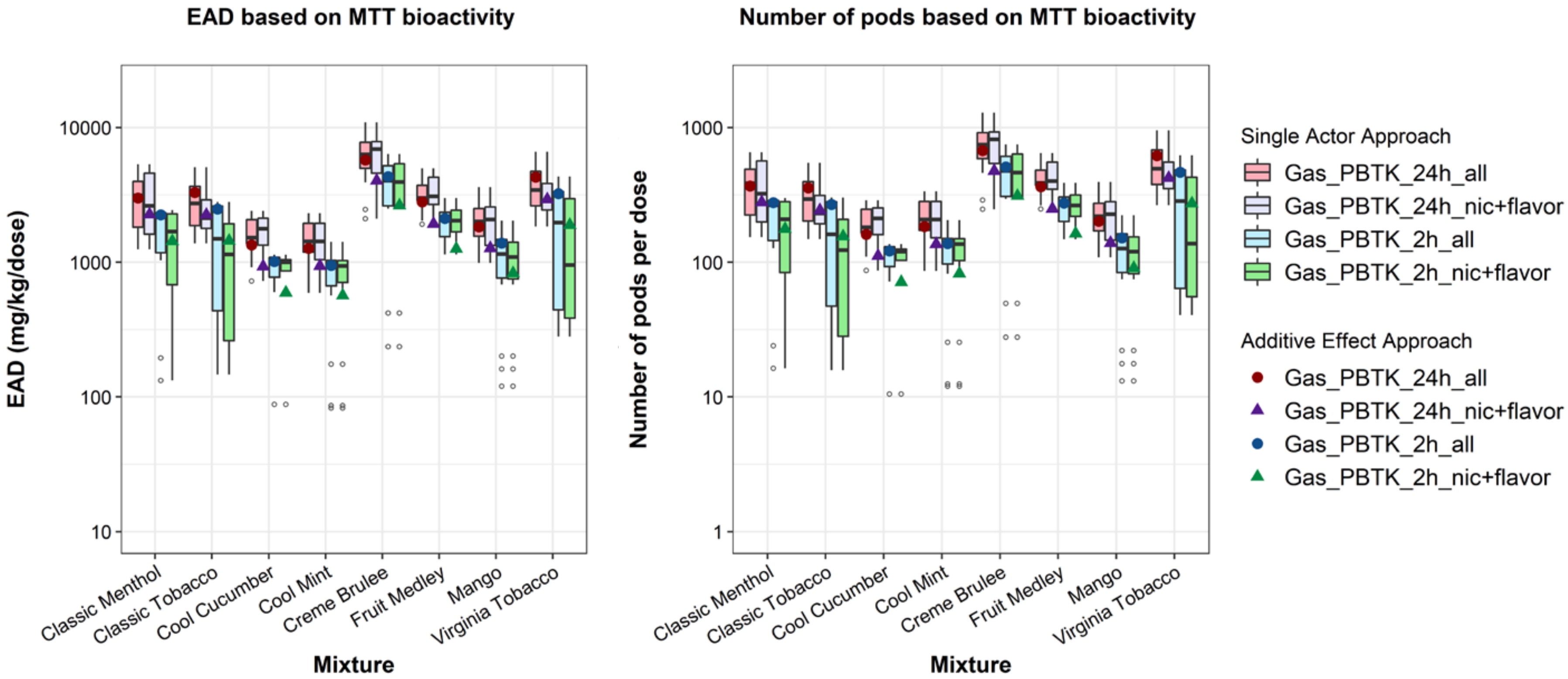

Supp. Figure S1B. EAD and pod comparison between inclusion and exclusion of carriers and BA, NRU assay, 24-hr and 2-hr interval, Gas_PBTK model

Figure S1B

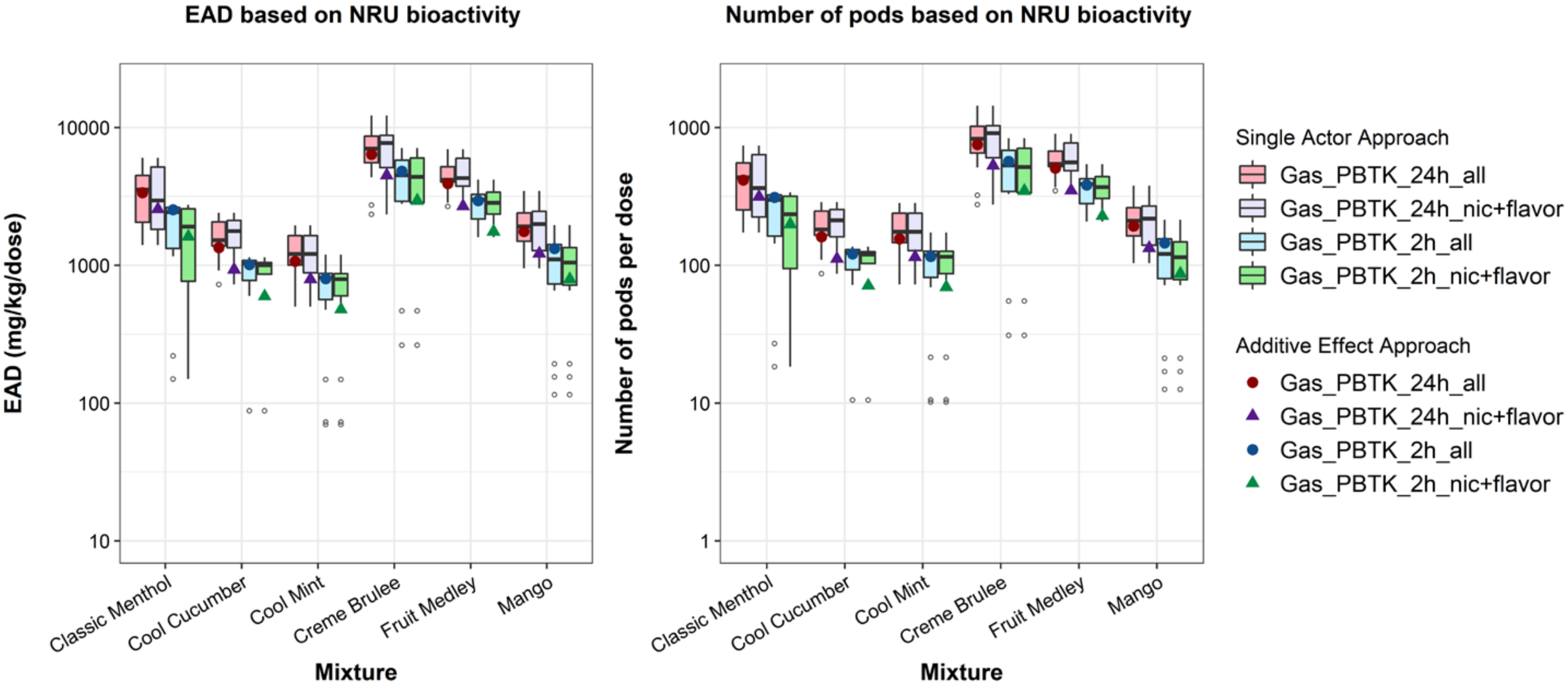

Supp. Figure S2A. EAD and pod comparison between inclusion and exclusion of carriers and BA, MTT assay, 24- and 2-hr interval, Solve_3C model with IV route

Figure S2A

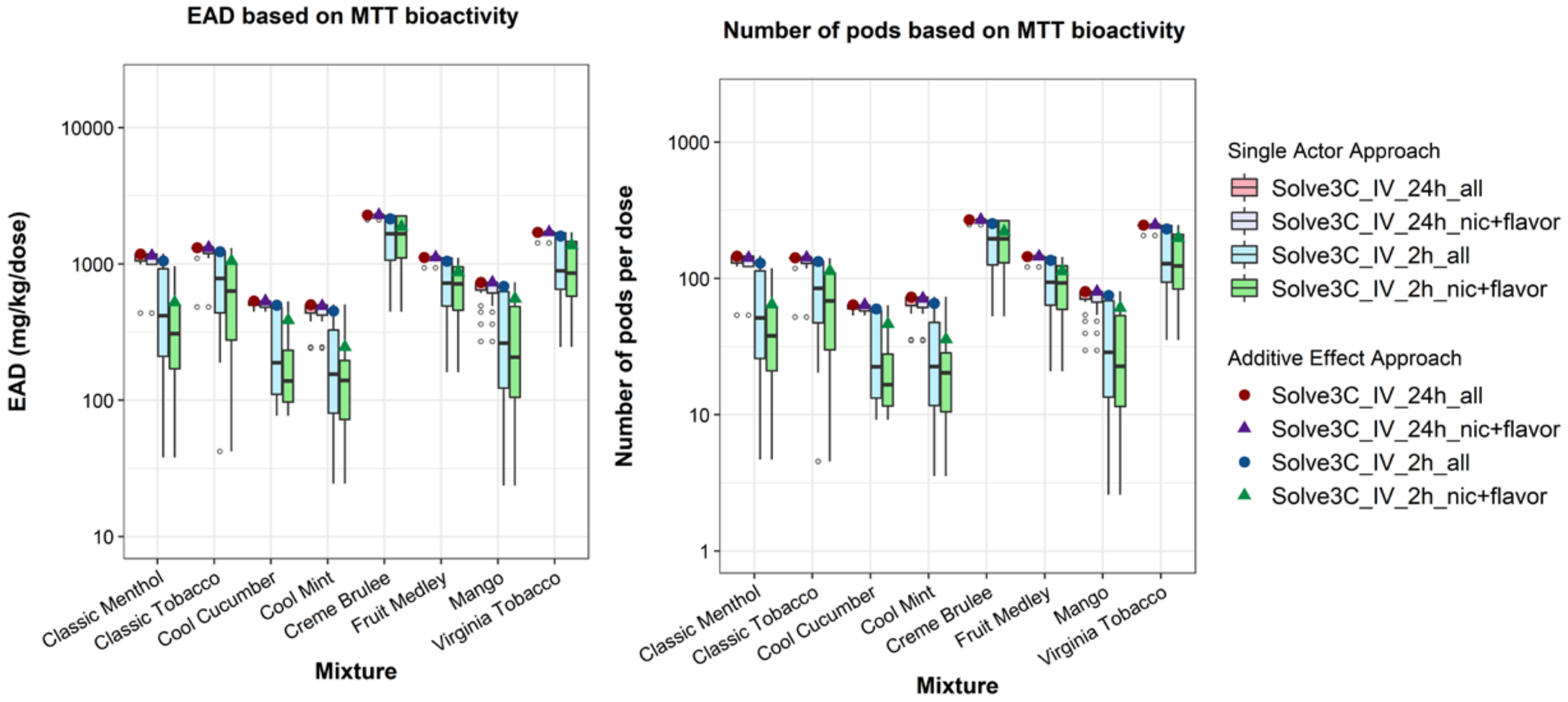

Supp. Figure S2B. EAD and pod comparison between inclusion and exclusion of carriers and BA, NRU assay, 24- and 2-hr interval, Solve_3C model with IV route

Figure S2B

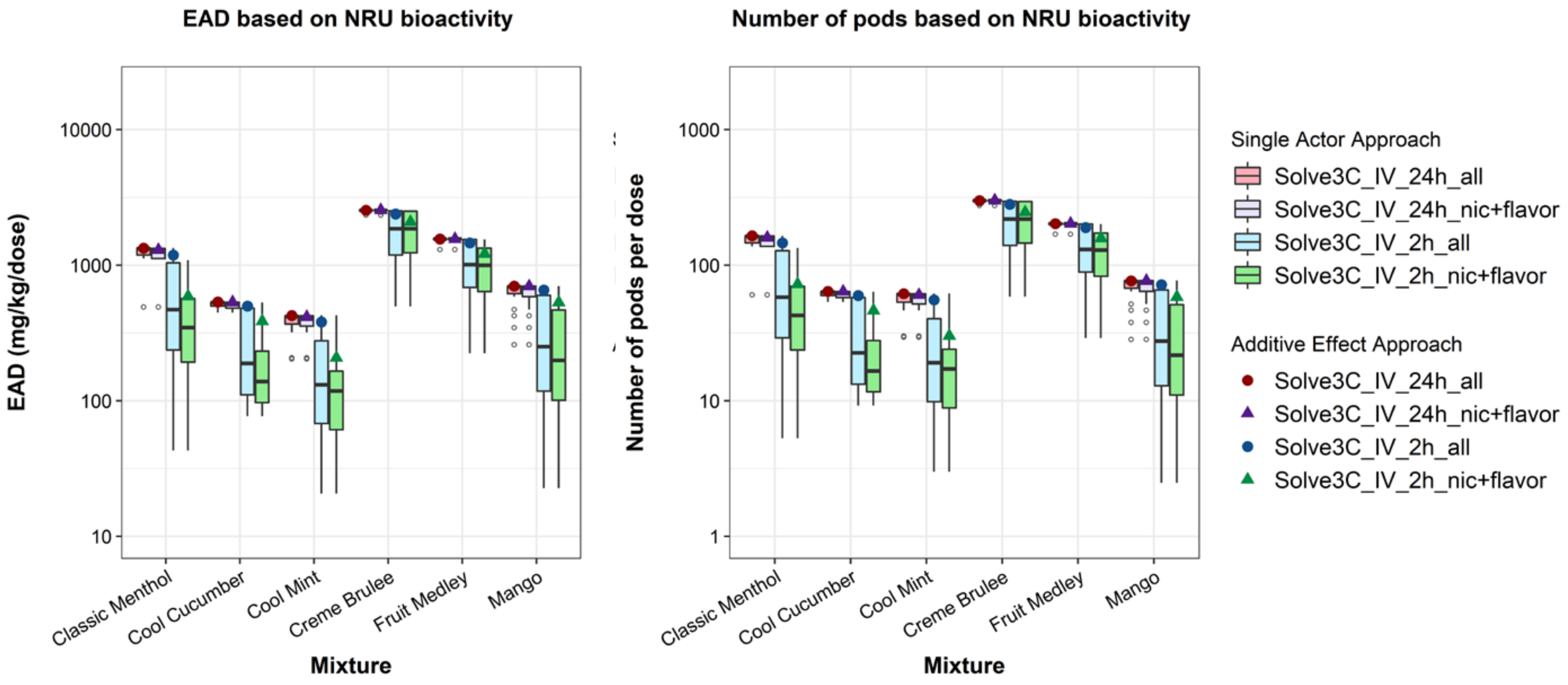

Supp Figure 3. Number of pods estimated using the $AC_{50}$ from the most sensitive cHTS assay using Gas_PBTK model

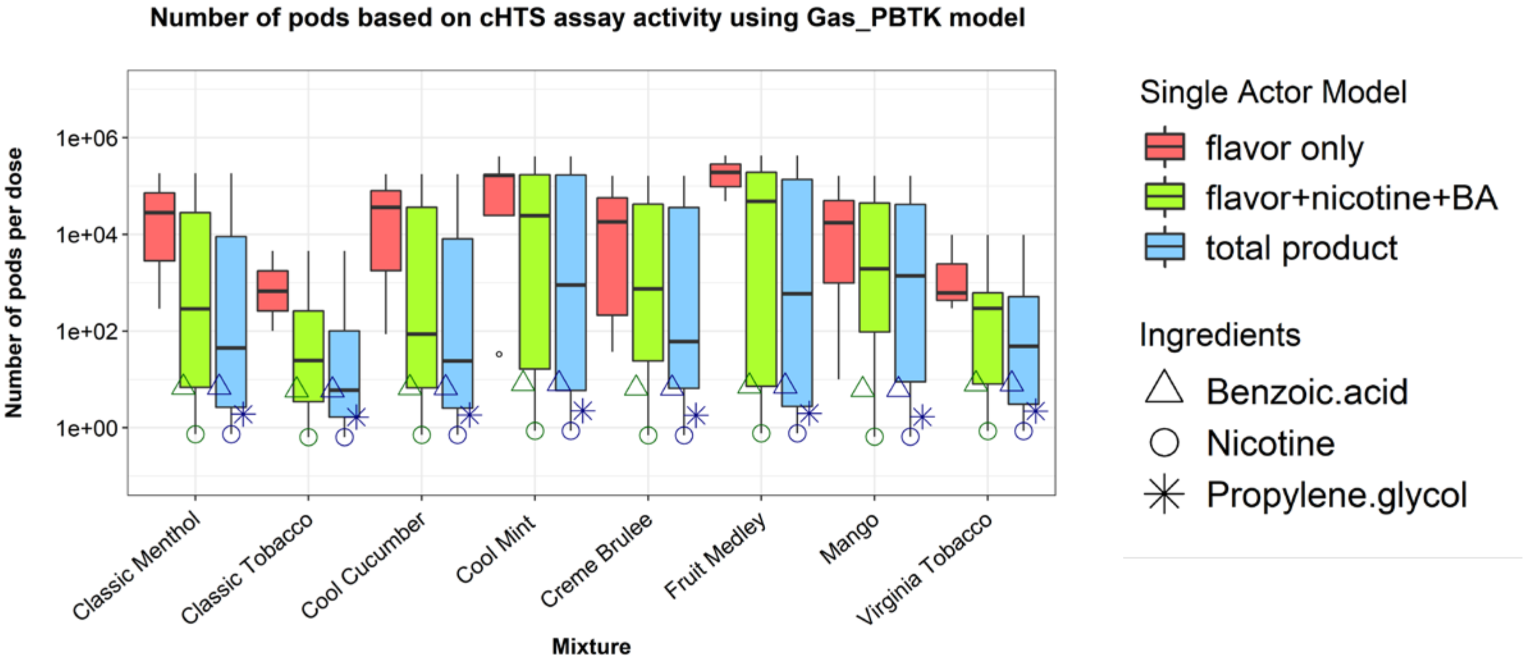


Supp. Figure 3. The n*umber of pods estimates for e-fluid flavors using the most potent HTS assay for individual ingredients. The data were plotted based on the following combinations: flavor only, flavor+nicotine+BA, and total product. The number of pods derived from bioactivity measured for Propylene glycol, Benzoic acid (BA) and nicotine are shown by various symbols. There were no HTS assays with bioactivity for glycerin in the dataset considered.*
